# Can artificial intelligence outperform experts in assessing clinical skills? Evidence from a comparative experiment

**DOI:** 10.3389/fmed.2026.1847867

**Published:** 2026-06-08

**Authors:** Yan Wang, Ning Wang

**Affiliations:** 1 Office of Academic Affairs, Nanjing Medical University, Nanjing, China; 2 Office of Teaching Management, Nanjing Medical University, Nanjing, China

**Keywords:** artificial intelligence, assessment, clinical skills, comparative experiment, medical education

## Abstract

**Background:**

This study evaluates and compares the performance of Artificial Intelligence Assessment (AIA) and Clinical Expert Assessment (CEA) in assessing clinical skills.

**Methods:**

Data were collected during two clinical skills competitions held in November 2024 and April 2025. Students’ skills were assessed on history-taking, endotracheal intubation, and cardio-pulmonary resuscitation (CPR) by CEA and AIA at the same time. Statistical analyses included intraclass correlation coefficient (ICC), Pearson’s correlation, paired-sample *t*-tests, and Bland-Altman plots.

**Results:**

For history-taking, endotracheal intubation, and CPR, scores of AIA and CEA showed moderate, excellent, and poor correlation, respectively. In history-taking, the results of ICC, Pearson’s correlation, and paired-sample *t*-tests (CEA-AIA) are 0.40, 0.57 and 8.23. In endotracheal intubation, the results are 0.92, 0.96 and −1.31. In CPR, the results are 0.20, 0.25 and 3.75.

**Discussion:**

The findings indicate that while AIA is good at objective structured content assessments, it struggles with subjective or context-rich scenarios. Conversely, CEA provides valuable holistic judgments but is likely to have bias and lack continuous attention. Combining both methods could leverage their respective strengths, improving evaluation reliability and educational outcomes.

**Conclusion:**

Integrating AIA for quantifying technical skills alongside CEA for comprehensive evaluation can enhance medical education assessments. Nonetheless, significant calibration and validation efforts are required before AI systems can be fully implemented.

## 1 Introduction

Clinical skills assessment is essential for competency-based medical education, particularly in the acquisition of clinical competencies, where trainees must demonstrate predetermined benchmark performance levels prior to advancing to subsequent training phases. Within China’s medical education framework, clinical skills assessment is predominantly the Objective Structured Clinical Examination (OSCE). In this standardized assessment model, students perform clinical skills within simulated scenarios while experienced clinicians conduct real-time scoring through observation.

However, using only two raters per skill station makes assessment results susceptible to clinical expert bias (1) and flaws due to lack of attention (2). Additionally, many Chinese medical universities enroll around 500 clinical students every year. Organizing clinical skills assessments for the students often requires approximately 1 week. For exam administrators, coordinating clinicians across multiple hospitals for assessments presents considerable logistical hurdles. It’s also limited to the need for experts with experience utilizing such metrics (1). Artificial intelligence (AI) may transform medical education assessment as we seek new technologies to reduce reliance on clinical experts.

Artificial intelligence employs computer programs to perform tasks traditionally requiring human execution. AI can identify patterns within large, complex datasets without being explicitly programmed. In assessment contexts, AI delivers rapid, real-time feedback and may detect performance patterns unrecognized by clinical experts.

Existing research primarily demonstrates the applicability of AI in medical education assessment. Applications of AI in Medical Education are mainly focusing on surgical skills training. AI accurately distinguishes expert from novice surgeons using kinematic data, surgical videos, and VR simulators and offers more objective, consistent, and real-time assessment than traditional subjective evaluation (3). AI has demonstrated strong validity in assessing surgical skills, narrative text, and clinical performance (4). Studies indicate that AI-based assessments of minimally invasive surgery skills exhibit variable accuracy from 63% to 100%, with Pearson correlation coefficients ranging from 0.60 to 0.85 (5). Other research reveals AI models achieve robust performance in simulated skill scenarios, demonstrating accuracy rates between 0.58 and 1.00 (6).

However, many studies display significant heterogeneity in the reliability of AI assessment (5). Systematically biased AI models reduced diagnostic accuracy (7). AI assessments exhibit reduced flexibility compared to clinical experts. Furthermore, unlike clinician bias affecting limited individuals, AI may risk introducing systemic biases. Such biases could disproportionately impact entire generations, racial groups, or gender populations, potentially exacerbating disparities among learner cohorts. Last but not least, many AI assessments are conducted in isolation, consequently lacking comparative validity evidence against clinical expert assessments (8).

This study aims to systematically compare the strengths and limitations of AI Assessment (AIA) and Clinical Expert Assessment (CEA) under standardized and comparable conditions, thereby providing more robust empirical evidence. Utilizing Kane’s validity framework, the study will conduct a comprehensive examination of the critical gaps in validity arguments for both AIA and CEA in medical education skills assessment. Through this investigation, we seek to establish an evidence-based foundation for the future implementation of AI-based assessment in medical education practice.

## 2 Materials and methods

This study employs Kane’s framework (9) to systematically organize, evaluate, and compare assessment scores of AIA and CEA. Kane’s framework, grounded in validation theory, serves as a methodological approach to comprehensively examine the scientific rigor and rational basis of assessment tools. The framework emphasizes argument-based validity, which constructs validity arguments through logical reasoning and empirical evidence rather than relying solely on singular statistical metrics. Kane’s framework evaluates assessment quality through four sequential inferences: scoring, generalization, extrapolation, and implications.

### 2.1 Analytical plan for Kane’s framework

The first step of Kane’s framework involves clarifying the intended interpretations and uses of the assessment. For AIA, the primary intended use is to assess clinical skills proficiency among medical students, provide formative feedback during training, and inform decisions regarding their transition from simulated training environments to real clinical settings.

The second step entails identifying the assumptions that support these interpretations and uses, organized according to Kane’s four inferences: scoring, generalization, extrapolation, and implications. We have identified a total of five key assumptions, summarized in Table 1. The list of assumptions acts as a hypothesis.

**TABLE 1:** Kane’s framework hypotheses.

Kane’s framework	Assumptions	Analyses
Scoring: Examines the relationship between observed performances and assigned scores, addressing whether the recorded scores accurately reflect students’ demonstrated competencies within the administered assessment context.	(1) The scoring rubric is rigorously validated, which can accurately reflect observed performance.	The scoring criteria system is derived from the nationally accredited textbook “Clinical Skills Operational Guidelines for Chinese Medical Students”, which has undergone official certification and is widely implemented across various clinical skills training programs in China.
Generalization: Determines if observed scores represent the student’s “universe score” – that is, whether they generalize across all potential observations within the defined domain (e.g., different scenarios, raters, or time).	(2) The assessment demonstrates high reproducibility. (3) The scores reliably represent the broader range of possible performances.	The inter-rater reliability between clinical experts was evaluated using intraclass correlation coefficient (ICC) to assess scoring consistency. AI scoring reproducibility was tested by comparing scores generated on the competition day with those produced 1 day later for the same dataset. The data collection process was optimized through randomized exam content, lottery-based participant selection, with examinees having no prior knowledge of the assessment content.
Extrapolation: Assesses whether observed scores provide predictive evidence for outcomes in authentic clinical environments.	(4) AIA scores demonstrate comparable accuracy to CEA in correctly reflecting students’ clinical competence levels.	The study examined the scoring correlation between AIA and CEA using ICC, Pearson’s correlation coefficient, paired-sample *t*-tests, and Bland-Altman analysis.
Implications: Evaluates how assessment-based decisions informed by observed scores lead to appropriate consequences for all stakeholders.	(5) AIA scoring results are appropriate for making valid assessments of students’ clinical proficiency.	The study conducted outlier analysis by reviewing on-site videos and correlating them with the assessment results from the expert review panel.

The most vulnerable and questionable hypotheses should be prioritized for analyzing. In the absence of prior empirical evidence, the analyses should follow the logical sequence from scoring to implications. Accordingly, hypotheses related to scoring (Hypothesis 1) are tested first, followed by reproducibility (Hypothesis 2) and validity (Hypothesis 3). These hypotheses take precedence because if they prove untenable, the remaining hypotheses would consequently fail. We developed a systematic plan to collect validity evidence that would either support or refute the proposed hypotheses. The evidence forms a validity argument supporting or challenging the intended interpretations and uses of both AIA and CEA.

### 2.2 Data collection

The data collection was carried out during two full-day sessions on November 19, 2024, and April 18, 2025. The two clinical skills competitions were held in Nanjing, Jiangsu Province, China. Student participants were voluntarily enrolled and included undergraduate and master’s students specializing in clinical medicine. The students came from 9 medical universities and 40 hospitals across China. Although institutions were widely sourced, participation was voluntary, and only a small number of students from each institution took part. The total valid sample was 60 students.

During the competitions, both clinical experts and an AI scoring system independently evaluated the same student performances, ensuring identical assessment targets and content. The scoring followed an OSCE format, assessing: history-taking, physical examination, internal medicine procedures, surgical procedures, emergency interventions.

For this study, basic information about data collection can be seen in Table 2. The endotracheal intubation is objective and highly structured. The history-taking is context-dependent and subjective. The CPR is a hybrid task. Additional technical specifications of the AI systems are detailed in Supplementary Material 1 of this paper.

**TABLE 2:** Basic information of data collection.

Time	Content	AI	Number of clinicians	Number of students
2024	History-taking	Self-developed AI assessment system	2	12
2024	Endotracheal intubation	Self-developed AI assessment system	2	24
2025	History-taking	Same as that of 2024	2	12
2025	Cardio-pulmonary resuscitation (CPR)	Self-developed AI assessment system	2	12

The competition results were not incorporated into formal academic evaluations or other critical assessments, thereby minimizing potential impacts on students’ educational progression and avoiding conflicts of interest. For the competition outcomes, only the expert-assigned scores were utilized, while the AI-generated scores were exclusively employed for validation and comparative analytical purposes. History-taking, endotracheal intubation, and cardio-pulmonary resuscitation (CPR)—each accounting for 3%–5% of the total score—were analyzed as research content. This study protocol received ethical approval from the Department of Academic Affairs of the University.

### 2.3 Rater

#### 2.3.1 History-taking AI assessment system

Students interact with a Virtual Standardized Patient in the system and input speech via the system. The system automatically generates history-taking transcripts through speech recognition software. The system’s technical framework follows a core closed-loop process of “interaction-simulation-reasoning-evaluation,” employing intelligent speech recognition technology to interact with students and collect data. It relies on deep integration of AI, VR, and medical knowledge, utilizing 3D modeling tools (Blender/Maya) to create high-fidelity virtual humans. Automatic Speech Recognition (ASR, e.g., Whisper) converts user speech to text, while Text-to-Speech (TTS, e.g., VITS) generates verbal responses from virtual patients. Large language models (e.g., GPT-4, Med-PaLM) enable dynamic responses to support open-ended questioning. Other detailed information about the system can be seen in Supplementary Material 1.

#### 2.3.2 Endotracheal intubation AI assessment system

This system provides comprehensive scoring by evaluating four dimensional metrics: spatial accuracy, operational fluency, time control, and overall coordination. It integrates three core technologies: (1) A multimodal sensing network combining inductance, pressure, and inertial sensors; (2) A hybrid communication protocol stack (LoRa + BLE + 5G multi-network coordination); and (3) A medical-specialized algorithm—a Spatiotemporal Convolutional Neural Network (ST-CNN) for performance evaluation. Other detailed information about the system can be seen in Supplementary Material 1.

#### 2.3.3 CPR AI assessment system

The system comprises a bipedal robot, a CPR manikin, and an AI analysis system. The bipedal robot adopts a reinforcement learning-based motion control strategy. The CPR manikin is equipped with chest compression sensors to capture depth and frequency data, along with oral/airway airflow sensors to measure ventilation volume and flow rate. The intelligent analysis system synchronizes real-time first-person-view footage from the robot, applying a pre-trained YOLO-Pose algorithm for skeletal keypoint extraction and body detection. For CPR performance evaluation, it calculates elbow joint angles and compares them against standardized threshold ranges. The system simultaneously integrates sensor data from the manikin to assess compression posture. Other detailed information about the system can be seen in Supplementary Material 1.

#### 2.3.4 Clinical expert

The assessments for history-taking, endotracheal intubation, and CPR were each evaluated by two clinical experts. The expert panels were composed of doctors recruited from different hospitals across Jiangsu Province, China. These doctors were selected through official work announcements. The doctors were systematically selected from relevant clinical disciplines that directly aligned with each assessed skill domain. All participating doctors held academic titles of associate professor (equivalent to deputy chief physician) or professor (chief physician), with 5–15 years of involvement in clinical medical education.

Prior to participation, all doctors underwent standardized faculty training along with specialized grading calibration for this particular competition, ensuring consistent and standardized evaluation competencies. Written informed consent was obtained from all participants.

#### 2.3.5 Arbitration panel

In cases of discrepancy between AIA and CEA scores, the contested results were referred to an independent arbitration panel for comprehensive analysis. The arbitration panel comprised three senior clinical medicine specialists recruited through official institutional announcements. All panel members held the academic rank of professor, with each possessing over 25 years of experience in medical education. Written informed consent was obtained from all participating arbitrators prior to their engagement in the review process.

### 2.4 Blinding measures

During the scoring process, any identifiable information was concealed to ensure that the experts remained unaware of students’ identities. Each assessment room was equipped with a corresponding AI evaluation system, which simultaneously scored the students’ skill demonstrations.

The clinical experts were not aware that they were being compared to an AI system. Before the competition, the experts were informed that the students had the right to appeal to the arbitration panel for verification of the scores. Therefore, no clinical expert raised any objection to the recording equipment.

Students wore uniformly provided surgical outerwear. Team and individual identification numbers, assigned by draw, were displayed on chest and back tags for recognition. Exchanging identification numbers during the competition was strictly prohibited, and all operations had to be performed exactly according to the assigned numbers specified in the test questions.

Throughout the competition, students were not permitted to disclose personal information such as their institution or name to the clinical experts, nor were they allowed to wear any clothing displaying institutional logos. Unauthorized communication with clinical experts was prohibited, and students could not ask anyone to clarify test questions. If issues or malfunctions arose, they were allowed to raise their hands to request assistance.

No early viewing of test questions or examination equipment was permitted. Technical support personnel were stationed in each examination room to address any AI system-related queries, though questions pertaining to test content were strictly prohibited.

### 2.5 Statistical analysis

Both the CEA and AIA employed identical assessment criteria and scoring rubrics. The final outputs from both were scores on a 0–100 scale, allowing for direct comparison without requiring score conversion or normalization.

Inter-rater reliability between the two clinical experts was assessed using the intraclass correlation coefficient (ICC). To evaluate the reproducibility of AI scoring, the AI system performed duplicate assessments on the same dataset - once during the competition day and again on the following day. Paired-sample *t*-tests were performed to examine mean differences between CEA and AIA. The Pearson correlation coefficient was calculated to determine the relationship between CEA and AIA. Measurement agreement and reliability were evaluated using ICC, followed by sensitivity and specificity analyses. A significance level of 0.05 was adopted for all statistical tests.

## 3 Result

The descriptive statistics of the collected data are presented in Table 3. During data processing, three obvious outliers with CEA-AIA value differences exceeding 30 were identified. Two of them were from the history-taking group, and one of them was from the CPR group. After review by the Arbitration Panel, these three clearly abnormal data points were approved for exclusion and will be used for subsequent outlier analysis. After excluding three outliers, all key results remained consistent with the analysis. We confirmed that exclusion of outliers did not change the core findings which confirms the stability of our conclusions.

**TABLE 3:** Descriptive Statistics of CEA and AIA.

Content	Score	Mean value	Standard deviation
History-taking	CEA	74.02	7.78
	AIA	65.80	10.60
Endotracheal intubation	CEA	66.94	17.45
	AIA	68.25	23.95
CPR	CEA	88.23	5.70
	AIA	84.48	4.35

We combined the 2-year history-taking data and conducted analyses on the three assessments: history-taking, endotracheal intubation, and CPR. Normality tests (Shapiro-Wilk) were performed on all three datasets, with results indicating that both the CEA and AIA scores for each assessment component followed normal distributions.

### 3.1 Evidence for scoring

The scoring criteria for both CEA and AIA were derived from the Chinese national textbook “Clinical Skills Operation Guide for Chinese Medical Students.” This authoritative guide was compiled by expert panels organized by the Center for Clinical Medical Education Research under China’s Ministry of Education. The manual explicitly defines the standardized operational competencies required for Chinese medical students and junior doctors.

As the official reference standard for the practical skills component of China’s National Clinical Physician Qualification Examination and a supplementary teaching material for standardized residency training programs, this guide has been widely adopted by medical schools across China. Its nationwide implementation and official endorsement ensure the scientific validity of the scoring system, which fully meets the research requirements of this study.

### 3.2 Evidence for generalization

In terms of CEA reliability test, we performed ICC analysis to assess the inter-rater reliability between the two clinical experts’ ratings in CEA. The correlation between the two experts’ scores for history-taking is 0.97. The correlation score in the endotracheal intubation assessment is 0.99. The correlation between the scores of experts in CPR assessment is 0.98. This exceptionally strong correlation indicates that both doctors provided nearly identical evaluations, further supporting the reliability of their judgment in assessments. Such a result is significant in indicating reliability in CEA scores.

In terms of AIA reliability test, we conducted scoring of the acquired dataset using the three AIA systems on the day after data collection. The AIA rescoring showed perfect reproducibility (ICC = 1.0, *p* < 0.05) between the two time points.

To make sure our simulation-based test was sufficient to assess students’ three skills, we used the “Clinical Skills Operation Guide for Chinese Medical Students” as the source of test content. The three skill assessment components each have their own specific criteria. The history-taking assessment evaluates the collection of fundamental patient information, including general data, present illness, past medical history, personal history, and family history, using electronic standardized patients. The endotracheal intubation is inserting a flexible plastic tube through a patient’s mouth or nose into the windpipe. The assessment focuses on spatial accuracy, procedural fluency, time management, and overall coordination. The CPR assessment examines the depth and frequency of chest compression, the volume of air delivered through the mouth, and the angle of the student’s elbow joint.

### 3.3 Evidence for extrapolation

This study compared CEA and AIA scoring across three clinical skills using four statistical methods. The methods are paired samples *t*-test, Pearson’s correlation test, the two-way random effects intraclass correlation coefficient (ICC) and the Bland-Altman plot analysis. History-taking revealed moderate correlation between CEA and AIA. Endotracheal intubation demonstrated excellent concordance. CPR assessment showed poor agreement. The brief results can be seen in Table 4 and detailed results analysis is presented below in this section.

**TABLE 4:** Agreement statistics between CEA and AIA.

Statistical method	Key metric	History-taking	Endotracheal intubation	CPR
Paired *t*-test	Mean difference (CEA-AIA)	+8.23**	−1.31*	+3.75*
	Effect size (Cohen’s d)	0.93 (Large)	−0.15 (small)	0.60 (medium)
Pearson’s correlation	Correlation coefficient (r)	0.57**	0.96**	0.25*
ICC	Single measures ICC	0.40**	0.92**	0.20*
Agreement assessment between CEA and AIA		Moderate	Excellent	Poor

**Means significance (*p* < 0.05);*means significance (*p* < 0.5).

#### 3.3.1 History-taking

In the history-taking assessment, paired samples *t*-test revealed that CEA scores were significantly higher than AIA scores by an average of 8.23 points (*p* < 0.05), with both statistical significance and practical importance (Cohen’s *d* = 0.93, indicating a large effect size). The two scoring methods demonstrated moderate positive correlation (*r* = 0.57, *p* = 0.005), suggesting that CEA employed notably more lenient standards than AIA.

Pearson’s correlation test confirmed this moderate-to-strong association between CEA and AIA.

The ICC analysis showed moderate-to-low association between CEA and AIA: single measures ICC = 0.40. The *F*-test indicated significant consistency (*p* = 0.003), demonstrating relatively stable and reliable agreement between raters.

**FIGURE 1:**
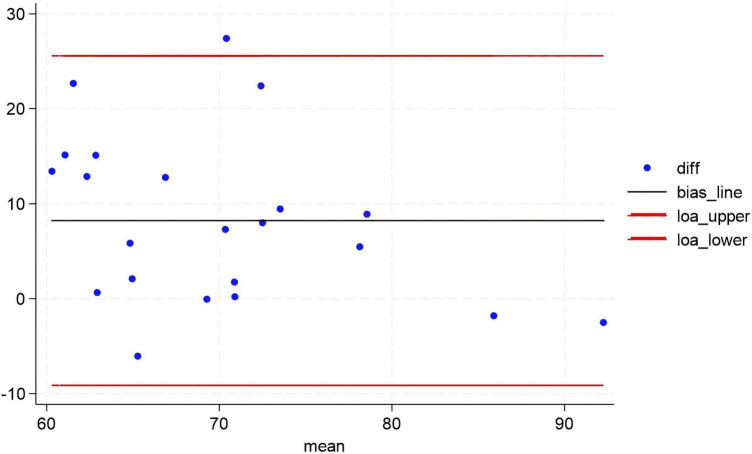
Bland-Altman plot of history-taking assessment.

The Bland-Altman plot compares CEA and AIA, with the horizontal axis showing mean scores and the vertical axis displaying differences (Figure 1). The black solid line represents the mean difference (bias) between the CEA and AIA. The upper red dashed line and lower red dashed line represent the 95% limits of agreement (LoA). The upper limit is calculated as bias +1.96 × standard deviation of the differences, and the lower limit is calculated as bias −1.96 × standard deviation of the differences. The red lines define a confidence interval for the differences between CEA and AIA. The LoA demonstrate that most data points fall within expected variation ranges, suggesting general method interchangeability. However, caution should be taken for values near the extremes (near 60 and near 90), where the differences between CEA and AIA are larger.

#### 3.3.2 endotracheal intubation

In the endotracheal intubation assessment, paired samples *t*-test demonstrated an exceptionally high correlation between CEA and AIA scores (*r* = 0.96, *p* < 0.05), indicating nearly identical scoring trends between the two methods. The *t*-test revealed that CEA on average is 1.31 points lower than AIA, though this difference was not very statistically significant (*p* = 0.468), with a 95% confidence interval [−4.97, 2.36] encompassing zero and demonstrating only minimal effect size (Cohen’s *d* = −0.15). Notably, AIA exhibited greater variability (SD = 23.95) compared to CEA (SD = 17.45), suggesting more fluctuation in AIA. During the scoring process, the AIA accurately scores based on the depth of tube insertion, whereas doctors can only observe with eyes and judge based on experience. This factor, to some extent, leads to greater variability in the AIA scoring results. Collectively, these findings reveal remarkable concordance between CEA and AIA.

Pearson’s correlation analysis confirmed an extremely strong association between CEA and AIA, establishing statistically significant alignment.

The ICC analysis showed excellent consistency: single measures ICC = 0.92 which substantially exceed the 0.75 benchmark for acceptability. The *F*-test (*p* < 0.05) confirmed the statistical significance of this high reliability.

**FIGURE 2:**
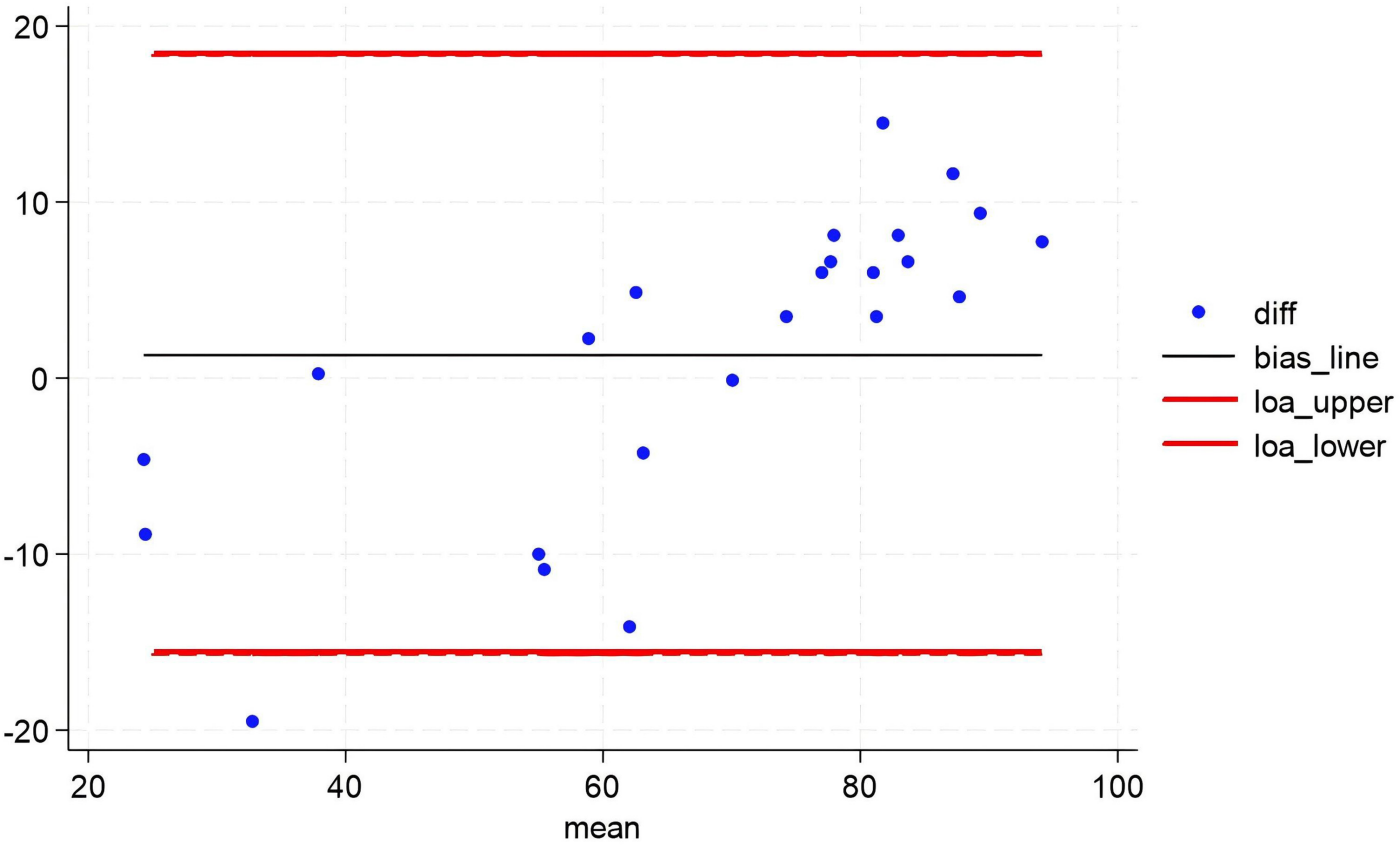
Bland-Altman plot of endotracheal intubation assessment.

The Bland-Altman analysis reveals minimal systematic bias between CEA and AIA (central line near zero), indicating comparable average performance (Figure 2). However, significant variability increases proportionally at higher score ranges. Most data points fall within the LoA. These findings support that CEA and AIA appear to perform similarly on average, but caution should be exercised when applying them to higher values, as the differences between CEA and AIA can become more pronounced.

#### 3.3.3 CPR

In the CPR assessment, paired samples *t*-test revealed that CEA scores were on average 3.75 points higher than AIA scores, though this difference did not reach statistical significance (*t* = 1.99, *p* = 0.075), with a moderate effect size (Cohen’s *d* = 0.60). The correlation between the two scoring methods was weak (*r* = 0.25, *p* = 0.47).

Pearson’s correlation analysis further confirmed the poor association between CEA and AIA, demonstrating neither statistical significance nor meaningful clinical correlation.

The ICC analysis showed unsatisfactory agreement between CEA and AIA: the absolute consistency ICC was merely 0.20 - substantially below the acceptable threshold of 0.5. The non-significant *F*-test result (*p* = 0.229) confirmed the absence of meaningful agreement between raters.

**FIGURE 3:**
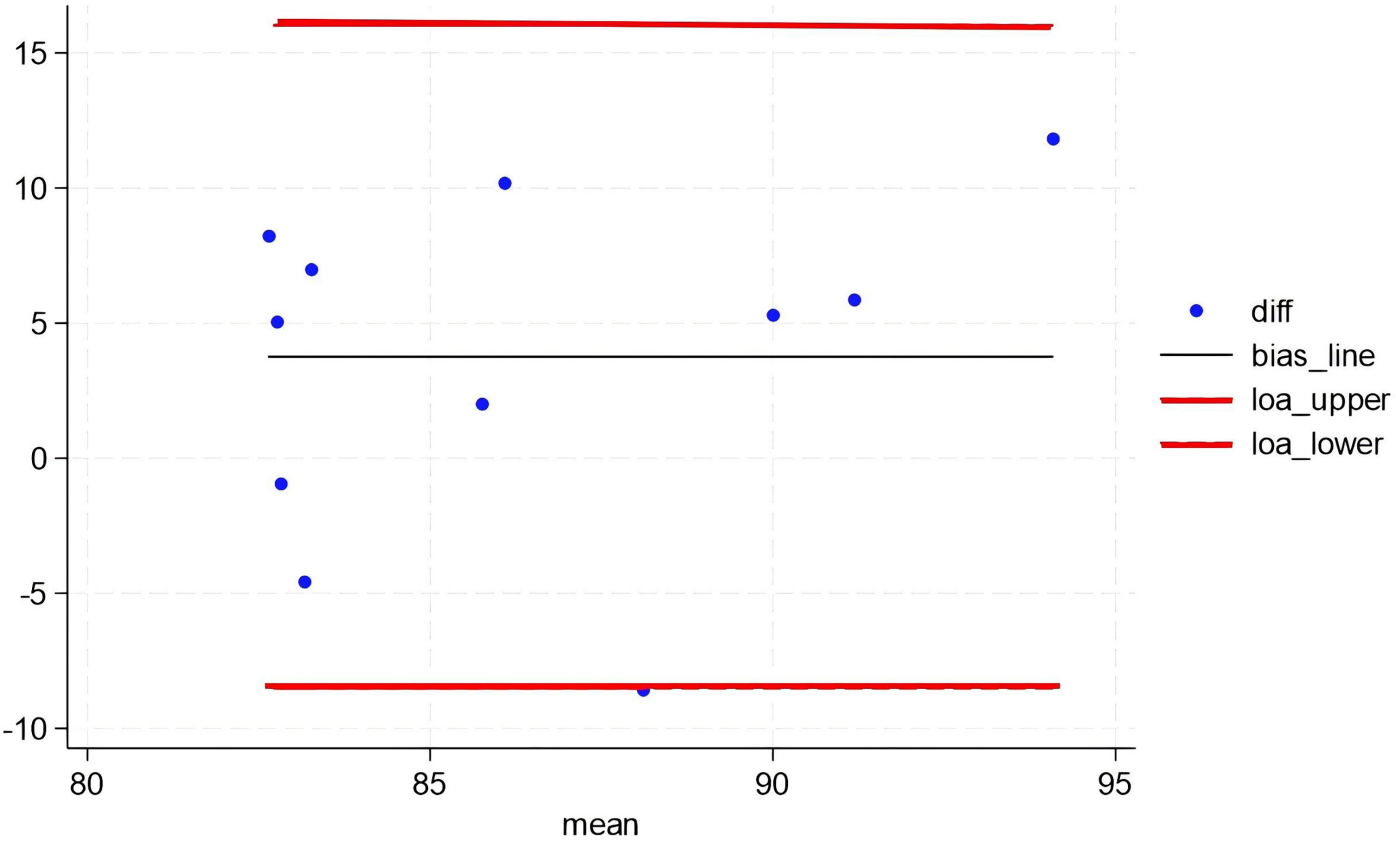
Bland-Altman plot of CPR.

The Bland-Altman analysis indicates generally good agreement between CEA and AIA, with minimal systematic bias (Figure 3). Most differences fall within the LoA, supporting method interchangeability. However, discrepancies are particularly evident for measurements at the higher end (near 90). While the methods demonstrate comparable performance on average, the increasing variability at upper ranges suggests potential proportional bias.

### 3.4 Evidence for implications

Taken together, these findings suggest that CEA and AIA exhibit moderate levels of agreement in their scoring patterns. The observed discrepancies likely reflect systematic differences in scoring stringency between CEA and AIA, while the correlation and reliability metrics indicate fundamental alignment in assessment frameworks. These results have important implications for the integration of AI-based assessment in medical education.

We defined potential outliers as cases where the discrepancy between CEA and AIA scores exceeded 30 points (out of 100 points). A 30-point gap represents a substantial performance tier difference (from incompetent to competent) in clinical skills assessment, which is educationally meaningful and likely reflects systematic scoring divergence rather than random variation. Besides, values beyond 30 points lie far outside the 95% limits of agreement in visual inspection of Bland-Altman plots, indicating abnormal discrepancies. These potential outliers were further evaluated through review of on-site videos and joint assessment by both arbitration panels and AI system engineers.

Two outliers in history-taking are caused by voice collection hardware issues affecting AI transcription completeness. In the history-taking assessment, we identified two outlier cases with score discrepancies of 70 and 32 points, respectively. Engineering analysis suggested these discrepancies likely resulted from hardware and environmental factors affecting voice data collection completeness, consequently impacting AIA scoring accuracy.

One outlier in CPR illustrated the difference shown in AI’s binary scoring and experts’ pedagogical judgment. For CPR assessment, one outlier case exhibited a 44-point discrepancy. Video review revealed the student’s arm flexion angle failed to meet technical standards, leading to an AI-determined score of zero compression. However, clinical experts awarded partial credit for demonstrated effort despite non-standardized performance, resulting in the significant scoring divergence. Supporting video evidence is provided in Supplementary Video 2.

When AI assesses advanced clinical competencies, its lack of interpretation may lead to “assessment avoidance”. Many educators reported rejecting AI recommendations due to incomprehensible scoring criteria. Our findings corroborate this phenomenon: in the CPR outlier case, while the student demonstrated the skill, the AI system’s binary pass-or-fail judgment on technical standards failed to recognize partial competency attainment. The all-or-nothing approach of AIA contradicts educational assessment principles and risks generating confusing feedback for both educators and learners. The observed discrepancies highlight critical challenges in aligning AI assessment with pedagogical evaluation standards.

## 4 Discussion

Following Kane’s framework, we categorized validity evidence in four aspects and identified critical distinctions in clinical skills assessment competency between CEA and AIA.

First, AI demonstrated significant advantages in objective structured content assessments. In the endotracheal intubation assessment, AI demonstrated superior performance compared to the other two systems. The reason is that the assessment criteria for endotracheal intubation are more amenable to standardization. This finding has been corroborated by other research. For instance, AI achieves higher accuracy in evaluating frequently used skills involving hand movements (e.g., suturing and knot-tying), while showing lower agreement with expert assessments in tasks requiring delicate visual judgment, such as tissue damage evaluation (10). In the assessment of bean drop and peg transfer, AI exhibited better performance in peg transfer due to its highly standardized movement patterns (11). Similarly, in robotic surgical skill evaluations, AI assessment results showed strong concordance with expert ratings (12). Consequently, multiple studies have confirmed that AI’s performance in structured tasks approaches expert-level assessment. These results suggest that AI could potentially alleviate some of the resource pressures in educational assessment systems in the future.

Secondly, although AI demonstrate significant advantages, certain limitations persist in their comprehensive behavioral assessment capabilities. AI-based evaluation is inherently confined to quantifiable technical performance rather than observational behavioral analysis. This discrepancy is exemplified by the heterogeneous scoring observed in CPR system assessments. AI performed poorly in CPR, a critical, dynamic, context-rich clinical skill. As evidenced by the on-site videos in Supplementary Video 2, distinct scoring disparities exist between AI and human raters. The AI system categorically assigns a “failed” rating if a candidate’s performance fails to meet predefined metrics, whereas clinical experts can discern deliberate attempts and assign partial credit accordingly. In medical education, procedural standards are established primarily through expert consensus as generalized guidelines for common clinical scenarios. However, real-world clinical practice involves far greater complexity, where therapeutic efficacy remains the sole definitive criterion. This represents an inherent gap between simulation-based training and clinical practice—a gap that AI cannot bridge. Human judgment remains indispensable for evaluating clinical treatment outcomes. Furthermore, studies indicate that AI systems underperform in tasks requiring multi-modal interaction due to their limited contextual comprehension and affective recognition capabilities (13). Literature has called for AI assessments that support rather than mimic the work of clinicians (14). Therefore, AI should be positioned as an adjunct rather than a replacement in medical education—a more rational direction for technological integration. For example, AI enables personalized skill training by delivering real-time quantitative feedback and actionable guidance during the instructional process (15).

Third, AI systems exhibited significant heterogeneity in competency and are considerably influenced by hardware variations. It’s necessary to comprehensively validate AI prior to clinical deployment. Although the history-taking system used in this study demonstrated a medium competency, the second time’s performance in 2025 was better than that of 2024. This indicates that after a period of training, the system used in this study achieved further performance optimization, leading to improved accuracy. In another study using a similar AI system, GPT-4o demonstrated a level of accuracy that was similar to that of senior experts in examining CPR skills examination videos (16). This suggests that large language models demonstrate strong potential for educational assessment. However, AI systems must undergo rigorous training and formal certification before being deployed in official educational evaluation settings.

This study has several limitations. First, the comparative experiments were conducted in a restricted scenario. To avoid potential negative impacts on student learning, data collection was limited to clinical skill competitions. The study was conducted in one Chinese city, using simulated competition settings. Only three clinical skills were tested. Findings cannot be directly generalized to other regions, or real clinical environments. We recommend future multi-center, multi-task, real-world educational studies to improve generalizability. Second, due to constraints in data acquisition, this study could not further validate whether AIA-generated results are suitable for educational feedback and related applications. Third, this study was a pilot comparative validation study of AI assessment in clinical skills. The results support the feasibility of AI assessment but require larger samples for definitive conclusions. We acknowledge in the Limitations that the small sample reduces statistical power and generalizability, and we recommend larger-scale studies in future.

## 5 Conclusion

Compared to CEA, AIA achieved excellent agreement only in highly standardized tasks, and it is not subject to issues such as human bias, fatigue, or lapses in attention. However, AIA has certain disadvantages in terms of situational comprehension and hardware requirements. Therefore, AIA cannot replace CEA in complex, holistic, or context-dependent assessment. Our research suggests that by leveraging the respective strengths of AIA and CEA—wherein AIA is responsible for quantitative evaluation of technical performance, while CEA contributes to the assessment of non-technical dimensions and overall judgment—a complementary scoring system can be established. This approach enhances both evaluation efficiency and overall reliability. Nevertheless, AI excels in well-defined, structured tasks because it relies on pattern matching rather than clinical or educational understanding. It cannot interpret student intent, while doctors recognize effort and give partial credit. Even as an auxiliary tool, human assessors must monitor for AI errors.

## Data Availability

The datasets presented in this study can be found in online repositories. The names of the repository/repositories and accession number(s) can be found in the article/Supplementary material.
