# Supplementary material for: Can artificial intelligence outperform experts in assessing clinical skills? Evidence from a comparative experiment
Source: Front Med (Lausanne). 2026 Jun 8;13:1847867. doi: 10.3389/fmed.2026.1847867 (PMC13283888; doi:10.3389/fmed.2026.1847867)
Supplement: Supplementary file 1 [file Data_Sheet_1.zip › supplement/1. AIA systems introduction/CPR AI assessment system introduction.docx]

Translation version：

I. System Description

The Intelligent CPR Evaluation System consists of a bipedal robot, a CPR training manikin, and an intelligent analysis system.

1. Bipedal Robot

Motion Control: Utilizes a reinforcement learning (RL)-based control scheme.

Training: Conducted in Nvidia's IsaacGym simulation environment and the legged_robot development framework.

Inference & Execution: Runs on an Intel N100 onboard computer.

Custom Mechanics: Combines gyroscope feedback, servo motor control, and RL reward-punishment algorithms to achieve stable bipedal walking.

Vision System:

Equipped with a 4MP dual-band (2.4G/5G) full-color PTZ binocular camera for 360° FOV monitoring.

Supports SD card recording and RTSP live streaming.

2. CPR Training Manikin

Specialized for CPR training, featuring:

Chest Compression Sensors: Measure depth and frequency of compressions.

Airflow Sensors: Installed in the mouth and airway to monitor flow rate and ventilation volume.

Independent Control Board & UPS Power System: Ensures stable operation.

Data Transmission: Supports Wi-Fi and serial communication.

3. Intelligent Analysis System

Real-Time Video Processing:

Synchronizes the robot’s first-person view via RTSP protocol.

Uses a pre-trained YOLO-Pose model for human detection and skeletal keypoint extraction.

Focuses on elbow joint angles for CPR evaluation:

Left Elbow Angle: Calculated using left wrist, elbow, and shoulder keypoints.

Right Elbow Angle: Similarly derived from right-side keypoints.

Compared against standard threshold ranges for scoring (other joints follow the same logic).

Sensor Data Integration:

Processes compression/ventilation data using Kalman filtering and state machine logic for phase segmentation.

Emphasizes posture evaluation during compressions.

Comprehensive Data Logging:

Records image data, posture metrics, compression/ventilation data, and evaluation results for in-depth analysis.


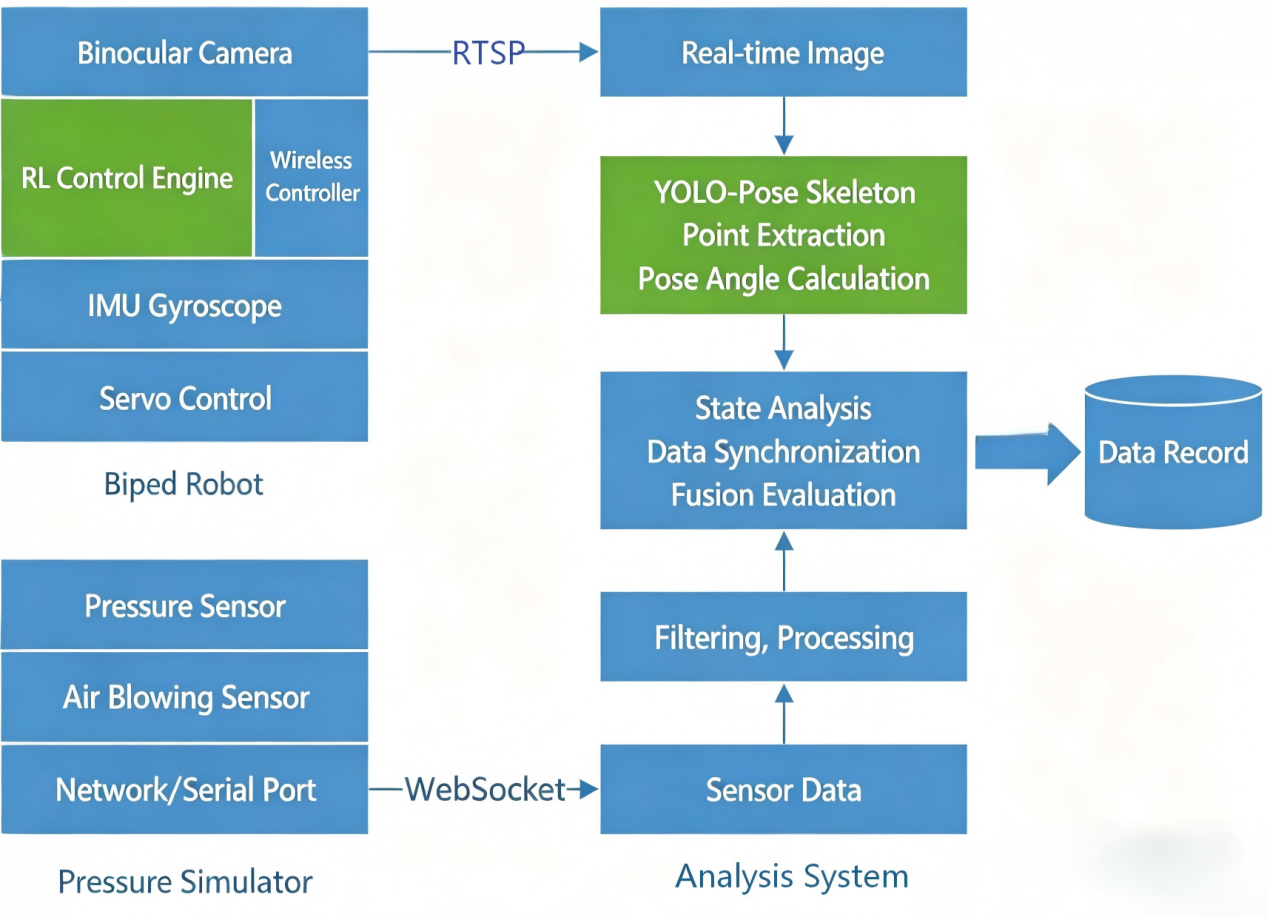


Figure 1

| Figure 1 translation from Chinese to English | |
| --- | --- |
| 双目摄像头 | Binocular Camera |
| R1控制引擎 | R1 Control Engine |
| 无线手柄 | Wireless Controller |
| IMU陀螺仪 | IMU Gyroscope |
| 伺服控制 | Servo Control |
| 双足机器人 | Bipedal Robot |
| 按压传感器 | Compression Sensor |
| 吹气传感器 | Ventilation Sensor |
| 网络/串口 | Network/Serial Port |
| RTSP | RTSP (Real Time Streaming Protocol) |
| 实时画面 | Live Feed |
| YOLO-Pose | YOLO-Pose |
| 骨骼点提取 | Skeletal Keypoint Extraction |
| 姿态角度计算 | Posture Angle Calculation |
| 状态分析 | State Analysis |
| 数据同步 | Data Synchronization |
| 融合评价 | Integrated Evaluation |
| 滤波、处理 | Filtering & Processing |
| WebSocket | WebSocket |
| 数据记录 | Data Logging |
| 传感器数据 | Sensor Data |
| 按压模拟人 | CPR Training Manikin |
| 分析系统 | Analysis System |


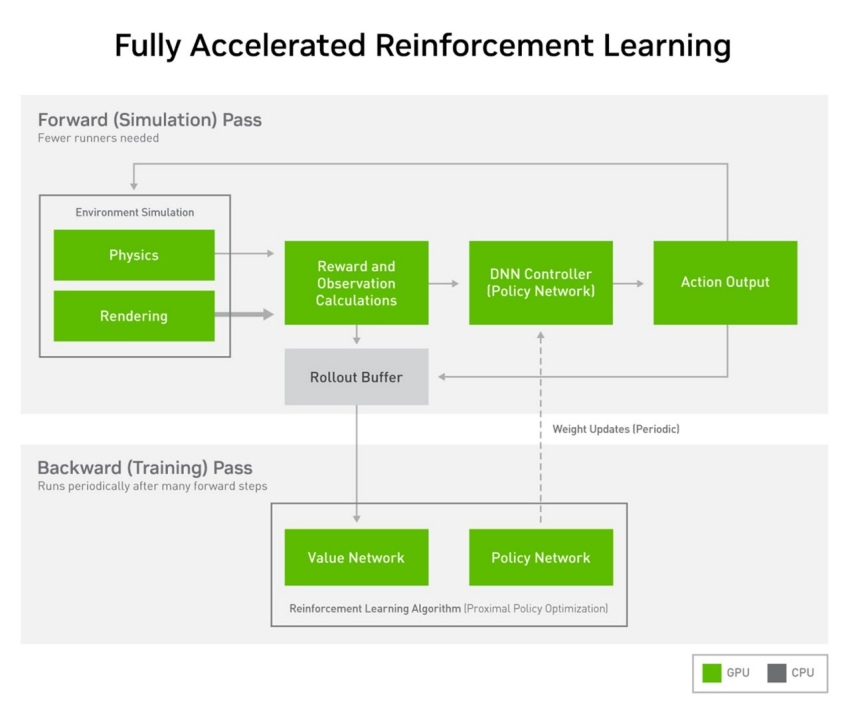


Figure 2 Reinforcement Learning Accelerated Workflow


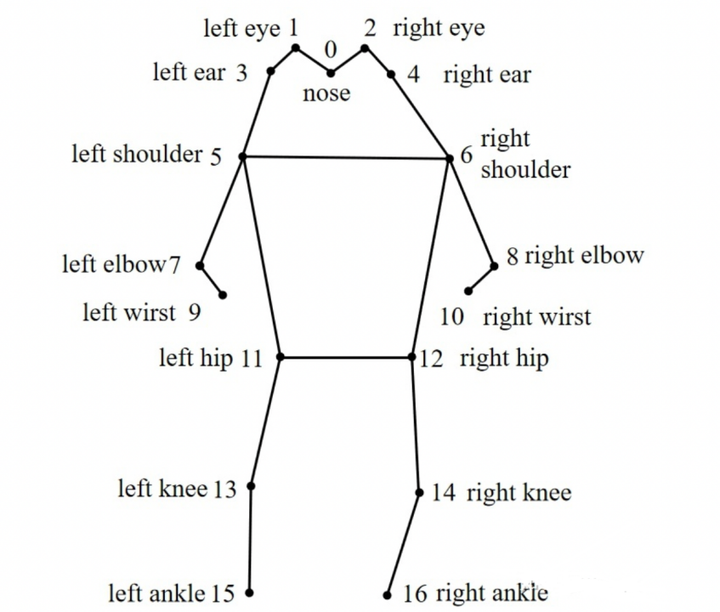


Figure 3 YOLO-Pose Key-points/Joints


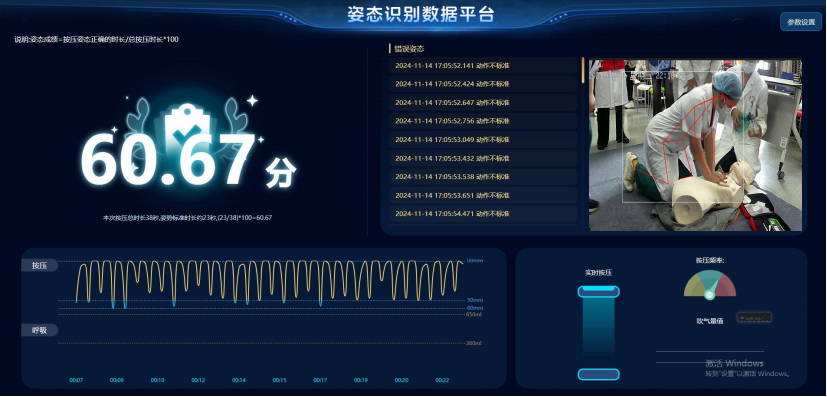


Figure 4 CPR system Front-end Interface

2: Record data interpretation

**2.1 CSV Posture File**

Each round of recording. The posture recognition is determined by judging the Right-angle and Left-angle values, which are both greater than 150 degrees to indicate the correct pressing posture.

| **key** | **name** |
| --- | --- |
| Right-shoulder-X | X-coordinate of the right shoulder |
| Right-shoulder-Y | Right shoulder y-coordinate |
| Right-shoulder-Z | Z-coordinate of the right shoulder |
| Right-elbow-X | X-coordinate of right elbow |
| Right-elbow-Y | Y-coordinate of the right elbow |
| Right-elbow-Z | Z-coordinate of the right elbow |
| Right-wrist-X | X-coordinate of the right wrist |
| Right-wrist-Y | Y-coordinate of the right wrist |
| Right-wrist-Z | Z-coordinate of the right wrist |
| Left-shoulder-X | Left shoulder x-coordinate |
| Left-shoulder-Y | Left shoulder y-coordinate |
| Left-shoulder-Z | Z-coordinate of the left shoulder |
| Left-elbow-X | X-coordinate of left elbow |
| Left-elbow-Y | Left elbow y-coordinate |
| Left-elbow-Z | Z-coordinate of the left elbow |
| Left-wrist-X | X-coordinate of left wrist |
| Left-wrist-Y | Y-coordinate of left wrist |
| Left-wrist-Z | Z-coordinate of the left wrist |
| Right-angle | The right arm is bent at an angle |
| Left-angle | The left arm is bent at an angle |

**2.2 JSON error log file**

Record each round. Record the image of the wrong posture and the time of pressing this time.

**2.3 TXT press sensor data**

Each round of records. The TXT file is a file that records the data of simulated human pressing sensors. The JSON field is described as follows:

| **key** | **field name** | **description** |
| --- | --- | --- |
| no | unused |  |
| DOWN | unused |  |
| LEFT | unused |  |
| fromserver | unused |  |
| ip | unused |  |
| groupid | unused |  |
| batch | unused |  |
| MID | unused |  |
| turn | unused |  |
| type | unused |  |
| mode | unused |  |
| DATATYPE | type | Press to indicate pressing |
| sceneType | unused |  |
| RIGHT | unused |  |
| planId | unused |  |
| UP | unused |  |
| TIMESTEP | unused |  |
| studId | unused |  |
| DEPTH | Press depth |  |
| datatime | Press the time point |  |

Some JSON messages have the same structure as blow messages, refer to section 2.4 Blow Data JSON. Press the following fields: press_avg_rate, press_depth_right_rate_statistic, press_pl_hm.

**2.4 Blowing data JSON**

All records are recorded in a file called Blowing Data.txt.

| **key** | **field name** | **description** |
| --- | --- | --- |
| no |  |  |
| press_depth_right_rate | Accuracy of pressing depth (percentage) |  |
| operate_right_rate | Operation accuracy (score) |  |
| operate_duration | Operation duration | millisecond |
| ls | The number of cycles, 5 is the full score | Press the blow cycle 5 times |
| press_right_rate | Press accuracy |  |
| cq_cs_pass_total | Number of blow-by passes |  |
| ay_cs_pass_total | Number of presses passed |  |
| ay_sd_sjz | unused |  |
| type | type | showData User display operation |
| cq_sd_total | Total volume of air blown | Ventilation Key Data Fields |
| operate_press_right_no | Number of correct operation press frequency |  |
| blowing_right_rate_statistic | unused |  |
| ay_sd_correct | unused |  |
| press_avg_rate | Average compression frequency (times/min) | Compression Key Data Fields |
| press_depth_right_rate_statistic | The ratio of the number of times the depth of compression is passed to the total number of times the compression is passed | Compression Key Data Fields |
| ay_zdz | Press the maximum value |  |
| press_pl | unused |  |
| ay_sjz | 1 indicates pressing |  |
| press_pl_hm | Press frequency | Compression Key Data Fields |
| press_rebound_right_rate | Press rebound accuracy (percentage) |  |
| ay_ht_yc | Abnormal pressing depth |  |
| ip | dummy man ip |  |
| batch | batch number |  |
| ay_zxz | Press the minimum value |  |
| cq_zdz | Blow up to the maximum |  |
| blowing_wrong_right | Blow-by error rate (percentage) |  |
| ls_press | number of epochs |  |
| ay_pl | unused |  |
| blowing_total_no | unused |  |
| cq_sd_now | Blow-by data | Ventilation Key Data Fields |
| currentTime | unused |  |
| ay_sd_yc | unused |  |
| cq_sd_yc | unused |  |
| ay_sd_jz | unused |  |
| press_total_no | Number of presses |  |
| blowing_right_rate | Blowing accuracy (percentage) |  |
| ay_sd_now | Press the depth in real time |  |
| cq_sd_jz | unused |  |
| time | Total duration (ms) |  |
| ay_pl_yc | Abnormal pressing frequency |  |
| press_blowing_rate | Pressurized air ratio (quantity ratio) |  |
| aytgcs | Number of presses passed |  |
| datatime | Current press time point |  |

Ventilation Key Data Fields: cq_sd_total, cq_sd_now.

Original version：

一：系统描述

心肺复苏智能评价系统由双足机器人、心肺复苏模拟人和智能分析系统组成。

双足机器人采用基于强化学习的运动控制方案，训练端使用Nvidia的IsaacGym仿真环境和legged_robot开发框架，选用Intel的N100主机在机器人侧进行推理和执行控制。针对自定义机械结构，结合陀螺仪数据反馈、伺服电机控制和RL奖惩算法实现机器人的双足行走。同时，机器人搭载400万双频(2.4G和5G)全彩双目云台摄像头，可实现360度全视野画面监测，并支持SD卡录制和RTSP实时流查看。

心肺复苏医疗模拟人为针对心肺复苏操作特别设计，胸部安装有按压传感器，支持按压深度、频率数据采集，口腔和气道连接吹气传感器，支持流速、吹气量测量。模拟人拥有独立控制主板和UPS电源系统，支持Wifi和串口等多种数据传输方式。

智能分析系统通过RTSP协议实时同步机器人第一视角画面，使用预训练的YOLO-Pose骨骼提取算法进行人体检测和骨骼关键点提取。对于心肺复苏操作，主要关注肘关节角度，因此，使用左腕、左肘和左肩骨骼点计算左肘关节角度，类似的，使用右腕、右肘和右肩骨骼点计算右肘关节角度，并与标准阈值区间进行比较评价，其他关节角度计算和评价与之类似。分析系统同时对接模拟人传感器数据，并使用卡尔曼滤波算法和状态机设计对操作数据进行处理和流程划分，且重点关注按压时的姿态评价。图像数据、姿态数据、按压数据、吹气数据和评价数据由系统实时记录，方便后续做深入分析。

图：心肺复苏评价系统框架


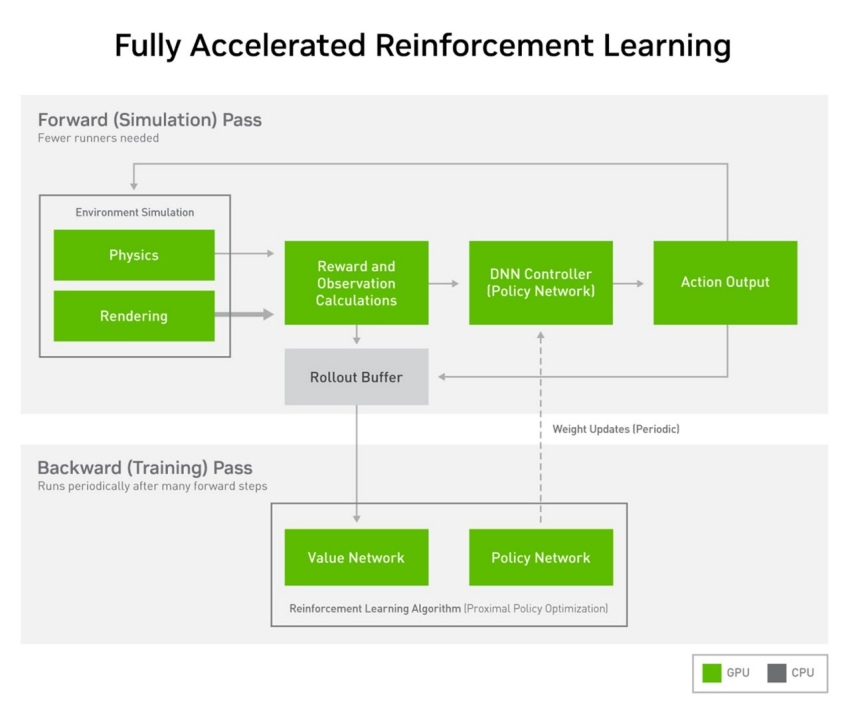


图2：强化学习加速工作流


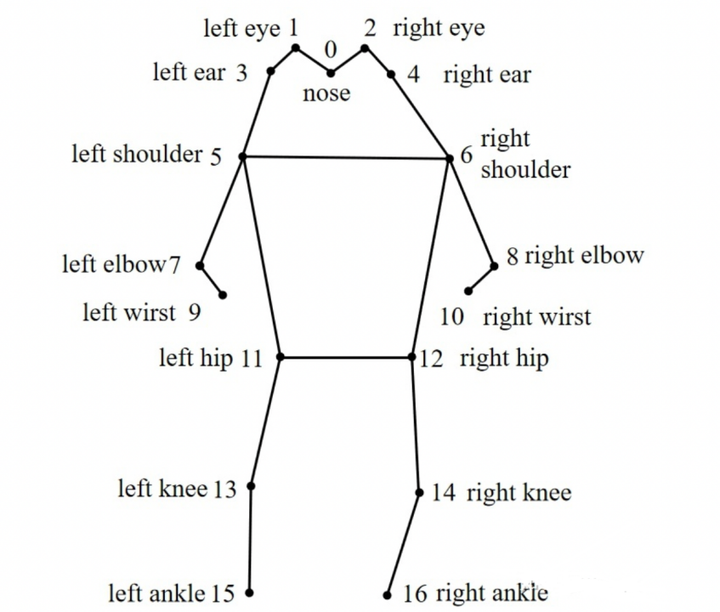


图3：TOLO-Pose关节点


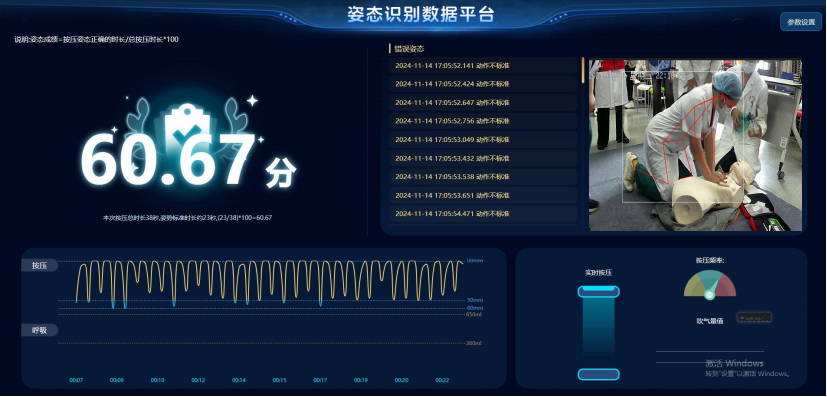


图：心肺复苏前端页面

二：记录数据解释

**2.1 CSV姿态文件**

每轮记录。姿态识别通过判断Right-angle和Left-angle值,均大于150度表示正确按压姿态。

| **key** | **名称** |
| --- | --- |
| Right-shoulder-X | 右肩x坐标 |
| Right-shoulder-Y | 右肩y坐标 |
| Right-shoulder-Z | 右肩z坐标 |
| Right-elbow-X | 右肘x坐标 |
| Right-elbow-Y | 右肘y坐标 |
| Right-elbow-Z | 右肘z坐标 |
| Right-wrist-X | 右手腕x坐标 |
| Right-wrist-Y | 右手腕y坐标 |
| Right-wrist-Z | 右手腕z坐标 |
| Left-shoulder-X | 左肩x坐标 |
| Left-shoulder-Y | 左肩y坐标 |
| Left-shoulder-Z | 左肩z坐标 |
| Left-elbow-X | 左肘x坐标 |
| Left-elbow-Y | 左肘y坐标 |
| Left-elbow-Z | 左肘z坐标 |
| Left-wrist-X | 左手腕x坐标 |
| Left-wrist-Y | 左手腕y坐标 |
| Left-wrist-Z | 左手腕z坐标 |
| Right-angle | 右手臂弯曲角度 |
| Left-angle | 左手臂弯曲角度 |

**2.2 JSON错误记录文件**

每轮记录。记录本次按压的错误姿态图片和按压时间。

**2.3 TXT按压传感器数据**

每轮记录。TXT文件为记录模拟人按压传感器数据的文件。JSON字段描述如下：

| **key** | **字段名** | **描述** |
| --- | --- | --- |
| no | 未使用 |  |
| DOWN | 未使用 |  |
| LEFT | 未使用 |  |
| fromserver | 未使用 |  |
| ip | 未使用 |  |
| groupid | 未使用 |  |
| batch | 未使用 |  |
| MID | 未使用 |  |
| turn | 未使用 |  |
| type | 未使用 |  |
| mode | 未使用 |  |
| DATATYPE | 类型 | PRESS表示按压 |
| sceneType | 未使用 |  |
| RIGHT | 未使用 |  |
| planId | 未使用 |  |
| UP | 未使用 |  |
| TIMESTEP | 未使用 |  |
| studId | 未使用 |  |
| DEPTH | 按压深度 |  |
| datatime | 按压时间点 |  |

部分JSON消息与吹气消息结构相同，参考《2.4 吹气数据JSON》部分。按压关注字段: press_avg_rate,press_depth_right_rate_statistic,press_pl_hm。

**2.4 吹气数据JSON**

全部记录在一份《吹气数据.txt》文件中。

| **key** | **字段名** | **描述** |
| --- | --- | --- |
| no |  |  |
| press_depth_right_rate | 按压深度准确率(百分比) |  |
| operate_right_rate | 操作准确率(得分数) |  |
| operate_duration | 操作持续时间 | 毫秒 |
| ls | 循环次数,5次为满分 | 按压吹气循环5次操作 |
| press_right_rate | 按压准确率 |  |
| cq_cs_pass_total | 吹气通过次数 |  |
| ay_cs_pass_total | 按压通过次数 |  |
| ay_sd_sjz | 未使用 |  |
| type | 类型 | showData用户显示操作 |
| cq_sd_total | 吹气量总数 | 吹气关注字段 |
| operate_press_right_no | 操作按压频率正确数 |  |
| blowing_right_rate_statistic | 未使用 |  |
| ay_sd_correct | 未使用 |  |
| press_avg_rate | 平均按压频率(次/分钟) | 按压关注字段 |
| press_depth_right_rate_statistic | 按压深度通过次数与按压总次数的比值 | 按压关注字段 |
| ay_zdz | 按压最大值 |  |
| press_pl | 未使用 |  |
| ay_sjz | 1表示按压中 |  |
| press_pl_hm | 按压频率 | 按压关注字段 |
| press_rebound_right_rate | 按压回弹正确率(百分比) |  |
| ay_ht_yc | 按压深度异常 |  |
| ip | 模拟人ip |  |
| batch | 批次号 |  |
| ay_zxz | 按压最小值 |  |
| cq_zdz | 吹起最大值 |  |
| blowing_wrong_right | 吹气错误率(百分比) |  |
| ls_press | 轮数 |  |
| ay_pl | 未使用 |  |
| blowing_total_no | 未使用 |  |
| cq_sd_now | 吹气数据 | 吹气关注字段 |
| currentTime | 未使用 |  |
| ay_sd_yc | 未使用 |  |
| cq_sd_yc | 未使用 |  |
| ay_sd_jz | 未使用 |  |
| press_total_no | 按压总次数 |  |
| blowing_right_rate | 吹气正确率(百分比) |  |
| ay_sd_now | 实时按压深度 |  |
| cq_sd_jz | 未使用 |  |
| time | 总时长(ms) |  |
| ay_pl_yc | 按压频率异常 |  |
| press_blowing_rate | 按压吹气比(量比) |  |
| aytgcs | 按压通过次数 |  |
| datatime | 当前按压时间点 |  |

吹气关注字段:cq_sd_total,cq_sd_now。

**注：评价系统不完善，需要补充专业评价量表描述。**
