# Supplementary material for: Can artificial intelligence outperform experts in assessing clinical skills? Evidence from a comparative experiment
Source: Front Med (Lausanne). 2026 Jun 8;13:1847867. doi: 10.3389/fmed.2026.1847867 (PMC13283888; doi:10.3389/fmed.2026.1847867)
Supplement: Supplementary file 1 [file Data_Sheet_1.zip › supplement/1. AIA systems introduction/endotracheal intubation AI assessment system introduction.docx]

Translated version：

**1.**

A training system for automatic evaluation of tracheal intubation operations based on the combination of embedded systems and three-dimensional visualization, characterized by comprising a tracheal model and an esophageal model that simulate human anatomical structures. The tracheal model includes the glottis, main trachea, left bronchus, and right bronchus. Metal detection sensors are installed at the glottis, main trachea, left main bronchus, right main bronchus, and esophagus to detect whether metal passes through their respective channels. The metal detection sensors are connected to an embedded control system, which transmits the detected information to the embedded control system.

**2.**

The training system for automatic evaluation of tracheal intubation operations based on the combination of embedded systems and three-dimensional visualization according to claim 1, characterized in that the embedded control system includes a main controller, a sensor control module, a network module, and a display module.

**3.**

The training system for automatic evaluation of tracheal intubation operations based on the combination of embedded systems and three-dimensional visualization according to claim 2, characterized in that the embedded controller connects to a backend server via a WIFI network for data exchange.

**4.**

The training system for automatic evaluation of tracheal intubation operations based on the combination of embedded systems and three-dimensional visualization according to claim 1, characterized in that during simulated tracheal intubation operations, the metal detection sensors detect and record data. The system analyzes which metal detection sensor the metal tube passes through. If it passes through the glottis or trachea, the result is judged as correct; if it passes through the main bronchus, the result is judged as overly deep insertion; if it passes through the esophagus, the result is judged as incorrect.

**5.**

The training system for automatic evaluation of tracheal intubation operations based on the combination of embedded systems and three-dimensional visualization according to claim 1, characterized in that the metal detection sensors determine whether they are triggered by detecting changes in inductance, thereby identifying the location of the intubation tube. The embedded control system obtains the trigger message of the metal detection sensors through pin interrupt events and transmits the intubation location data to the server. The server controls the three-dimensional application to play different animations, achieving intuitive and timely visual feedback.

**6.**

The training system for automatic evaluation of tracheal intubation operations based on the combination of embedded systems and three-dimensional visualization according to claim 1, characterized in that plastic materials are used to simulate the glottis, trachea, main bronchi, and esophagus, while silicone materials are used to simulate skin and muscle tissues, with the overall structure mimicking the basic human anatomy.

**7.**

A training method for automatic evaluation of tracheal intubation operations based on the combination of embedded systems and three-dimensional visualization, characterized by the following steps:

Step 1: Start the camera and adjust its angle to fully capture the operating area.

Step 2: Open the web-based program, log into the three-dimensional tracheal intubation training platform using an account and password, and verify that the camera feed is functioning properly.

Step 3: Activate the embedded controller on the tracheal intubation simulator.

Step 4: After completing the above steps, click the start button on the page to begin training. During the operation, the metal detection sensors installed at the glottis, trachea, left main bronchus, right main bronchus, and esophagus will detect the location of the metal intubation tube. The embedded controller then transmits the current information to the backend server via the network.

Step 5: After each training session, statistical data is recorded, and the training video is automatically saved for later playback.

**8.**

The training method for automatic evaluation of tracheal intubation operations based on the combination of embedded systems and three-dimensional visualization according to claim 7, characterized by differentiating the materials of the model and the intubation tube to create conditions for the metal detection sensors to detect the tube.

**9.**

The training method for automatic evaluation of tracheal intubation operations based on the combination of embedded systems and three-dimensional visualization according to claim 7, characterized by using metal detection sensors to detect the intubation tube and determine its specific location within the model.

**10.**

The training method for automatic evaluation of tracheal intubation operations based on the combination of embedded systems and three-dimensional visualization according to claim 9, characterized by the following evaluation criteria based on the operation results:

(1) Glottis triggered: Indicates the tube has entered the main trachea, a normal operation.

(2) Trachea triggered: The tube is located in the main trachea, a normal operation.

(3) Left or right main bronchus triggered: The tube has passed the main trachea and entered the bronchus, an overly deep insertion.

(4) Esophagus triggered: The tube has entered the esophagus instead of the main trachea, an incorrect operation.

---

**A Training System and Method for Automatic Evaluation of Tracheal Intubation Operations Based on the Combination of Embedded Systems and Three-Dimensional Visualization**

**Technical Field**

The invention relates to the field of medical education and practical training, specifically a training system and method for automatic evaluation of tracheal intubation operations based on the combination of embedded systems and three-dimensional visualization.

**Background Technology**

Traditional tracheal intubation training relies on professional instructors for on-site evaluation. Instructors use their experience to assess the correctness of the trainee's tube placement, force, depth, and speed. However, this training method has the following five issues:

(1) High dependence on instructors and their experience.

(2) Difficulty in quantifying training outcomes.

(3) Monotonous training with no interaction, leading to low trainee engagement.

(4) Limited to classroom settings, restricting access to instructor guidance due to time and space constraints.

(5) For tracheal intubation, instructors cannot directly observe the correctness of the operation due to the model's concealment.

With advancements in technology, the maturity of 3D simulation, and the modularization of embedded systems, interdisciplinary integration has gained attention, showing great potential in medical education. However, there is currently a lack of training systems that combine 3D simulation and embedded technology.

**Invention Content**

The purpose of the invention is to provide a training system and method for automatic evaluation of tracheal intubation operations based on the combination of embedded systems and three-dimensional visualization, improving training efficiency and effectiveness.

To achieve this goal, the technical solution of the invention is as follows:

On one hand, the invention provides a training system for automatic evaluation of tracheal intubation operations based on the combination of embedded systems and three-dimensional visualization. The system includes a tracheal model and an esophageal model that simulate human anatomical structures. The tracheal model includes the glottis, main trachea, left bronchus, and right bronchus. Metal detection sensors are installed at the glottis, main trachea, left main bronchus, right main bronchus, and esophagus to detect whether metal passes through their respective channels. The sensors are connected to an embedded control system, which transmits the detected information to the embedded control system.

Further, the embedded control system includes a main controller, a sensor control module, a network module, and a display module.

Moreover, the embedded controller connects to a backend server via a WIFI network for data exchange.

Further, during simulated tracheal intubation operations, the metal detection sensors detect and record data. The system analyzes which metal detection sensor the metal tube passes through. If it passes through the glottis or trachea, the result is judged as correct; if it passes through the main bronchus, the result is judged as overly deep insertion; if it passes through the esophagus, the result is judged as incorrect.

Further, the metal detection sensors determine whether they are triggered by detecting changes in inductance, thereby identifying the location of the intubation tube. The embedded control system obtains the trigger message of the metal detection sensors through pin interrupt events and transmits the intubation location data to the server. The server controls the three-dimensional application to play different animations, achieving intuitive and timely visual feedback.

Further, plastic materials are used to simulate the glottis, trachea, main bronchi, and esophagus, while silicone materials are used to simulate skin and muscle tissues, with the overall structure mimicking the basic human anatomy.

On the other hand, the invention also provides a training method for automatic evaluation of tracheal intubation operations based on the combination of embedded systems and three-dimensional visualization. The training steps are as follows:

Step 1: Start the camera and adjust its angle to fully capture the operating area.

Step 2: Open the web-based program, log into the three-dimensional tracheal intubation training platform using an account and password, and verify that the camera feed is functioning properly.

Step 3: Activate the embedded controller on the tracheal intubation simulator.

Step 4: After completing the above steps, click the start button on the page to begin training. During the operation, the metal detection sensors installed at the glottis, trachea, left main bronchus, right main bronchus, and esophagus will detect the location of the metal intubation tube. The embedded controller then transmits the current information to the backend server via the network.

Step 5: After each training session, statistical data is recorded, and the training video is automatically saved for later playback.

Further, the method involves differentiating the materials of the model and the intubation tube to create conditions for the metal detection sensors to detect the tube.

Further, the method uses metal detection sensors to detect the intubation tube and determine its specific location within the model.

Moreover, the evaluation criteria based on the operation results are as follows:

(1) Glottis triggered: Indicates the tube has entered the main trachea, a normal operation.

(2) Trachea triggered: The tube is located in the main trachea, a normal operation.

(3) Left or right main bronchus triggered: The tube has passed the main trachea and entered the bronchus, an overly deep insertion.

(4) Esophagus triggered: The tube has entered the esophagus instead of the main trachea, an incorrect operation.

**Advantageous Effects**

The invention enables medical personnel to intuitively judge the correctness and standardization of their operations, achieving the goal of training. The system realizes the detection of key intubation positions and combines with three-dimensional visualization to provide intuitive and timely feedback.

**Brief Description of the Drawings**

Figure 1 is a schematic diagram of the training system for automatic evaluation of tracheal intubation operations.

Figure 2 is a flowchart of the training method for automatic evaluation of tracheal intubation operations.

**Detailed Description of the Embodiments**

The following describes the invention in detail with reference to the accompanying drawings and specific embodiments.

**Embodiment 1**

As shown in Figure 1, the training system includes a tracheal model and an esophageal model that simulate human anatomical structures. The tracheal model includes the glottis, main trachea, left bronchus, and right bronchus. Plastic materials are used to simulate the glottis, trachea, main bronchi, and esophagus, while silicone materials are used to simulate skin and muscle tissues, with the overall structure mimicking the basic human anatomy. Metal detection sensors are installed at the glottis, main trachea, left main bronchus, right main bronchus, and esophagus to detect whether metal passes through their respective channels. The sensors are connected to an embedded control system, which transmits the detected information to the embedded control system.

The embedded control system includes a main controller, a sensor control module, a network module, and a display module. The embedded controller connects to a backend server via a WIFI network for data exchange. During simulated tracheal intubation operations, the metal detection sensors detect and record data. The system analyzes which metal detection sensor the metal tube passes through. If it passes through the glottis or trachea, the result is judged as correct; if it passes through the main bronchus, the result is judged as overly deep insertion; if it passes through the esophagus, the result is judged as incorrect.

The metal detection sensors determine whether they are triggered by detecting changes in inductance, thereby identifying the location of the intubation tube. The embedded control system obtains the trigger message of the metal detection sensors through pin interrupt events and transmits the intubation location data to the server. The server controls the three-dimensional application to play different animations, achieving intuitive and timely visual feedback.

**Embodiment 2**

As shown in Figure 2, the training method includes the following steps:

Step 1: Prepare the training recording camera. Start the camera and adjust its angle to fully capture the operating area.

Step 2: Open the web-based program, log into the training platform using an account and password. Upon entering the three-dimensional tracheal intubation training platform, the camera feed and the initial 3D animation state of the tracheal intubation can be viewed.

Step 3: Activate the embedded controller on the tracheal intubation simulator. For first-time use, press and hold the reset button for five seconds to enter the reset program.

- Open the WIFI on the phone and search for a hotspot network named "ESP_ETT." Connect to this network.

- After successful connection, open the browser on the phone and enter "http://esp_ett.local" to access the configuration page. Enter the available WIFI name (no Chinese characters), password, and the deployed SOCKET service address, then click submit. The embedded system will then connect to the WIFI and backend service.

- The TFT LCD screen on the embedded controller displays the system's operational status. If the embedded system fails to establish a network connection with the server, check the current network environment or reconfigure the settings to ensure subsequent steps proceed smoothly.

Step 4: After completing the above steps, click the start button on the page to begin training. During the operation, the sensors will identify the intubation location, and the webpage will display the corresponding animation and insertion site.

The operation involves embedding induction modules in the plastic and silicone models simulating the glottis, trachea, left and right main bronchi, and esophagus, along with the metal core of the intubation tube. The metal detection sensors prompt data collection and recording when metal passes through their respective channels. The sensors are connected to the embedded control system, which exchanges data with the backend server via the WIFI network. During simulated tracheal intubation operations, the metal detection sensors detect and record data. The system analyzes which metal detection sensor the metal tube passes through.

The metal detection sensors determine whether they are triggered by detecting changes in inductance. The embedded system uses the interrupt feature of the pins to update the detection status when the sensor's output level changes and transmits the current data to the backend server via the established network. The 3D simulation program pre-creates animations corresponding to triggers at the glottis, trachea, left main bronchus, right main bronchus, and esophagus. When the embedded system notifies a status update, the 3D simulation program plays the corresponding animation, providing intuitive and timely training feedback.

The evaluation criteria based on the operation results are as follows:

(1) Glottis triggered: Indicates the tube has entered the main trachea, a normal operation.

(2) Trachea triggered: The tube is located in the main trachea, a normal operation.

(3) Left or right main bronchus triggered: The tube has passed the main trachea and entered the bronchus, an overly deep insertion.

(4) Esophagus triggered: The tube has entered the esophagus instead of the main trachea, an incorrect operation.

Step 5: After each training session, the data is recorded and saved. The training video is automatically saved for later playback.

In the above process, the system achieves detection of key intubation positions: Step 4 uses the metal intubation tube and metal detection sensors. When the metal tube passes through the sensors installed at the glottis, trachea, left main bronchus, right main bronchus, and esophagus, it causes changes in the sensors' inductance. The embedded controller captures the trigger status of the sensors through pin interrupts, thereby determining the intubation location.

The above process also achieves the integration of the embedded system with 3D visualization: Step 4 uses metal detection sensors and the embedded controller to obtain the operational status of the tracheal intubation. The data is transmitted via the network to interact with the 3D application, which plays different operational animations.

In summary, the invention realizes the transmission of sensing data for key points such as position, force, depth, and speed during intubation, which are not directly observable. It connects with the 3D simulation model for interactive visualization and enables automatic evaluation based on assessment points such as position, force, depth, and speed, ultimately achieving quantifiable, standardized, and normalized operational assessments.

The above description outlines the basic principles, main features, and advantages of the invention. Those skilled in the art should understand that the embodiments do not limit the scope of the invention in any form. Technical solutions obtained through equivalent substitutions or other means fall within the scope of the invention.

Parts of the invention not mentioned are identical to or can be implemented using existing technologies.

---


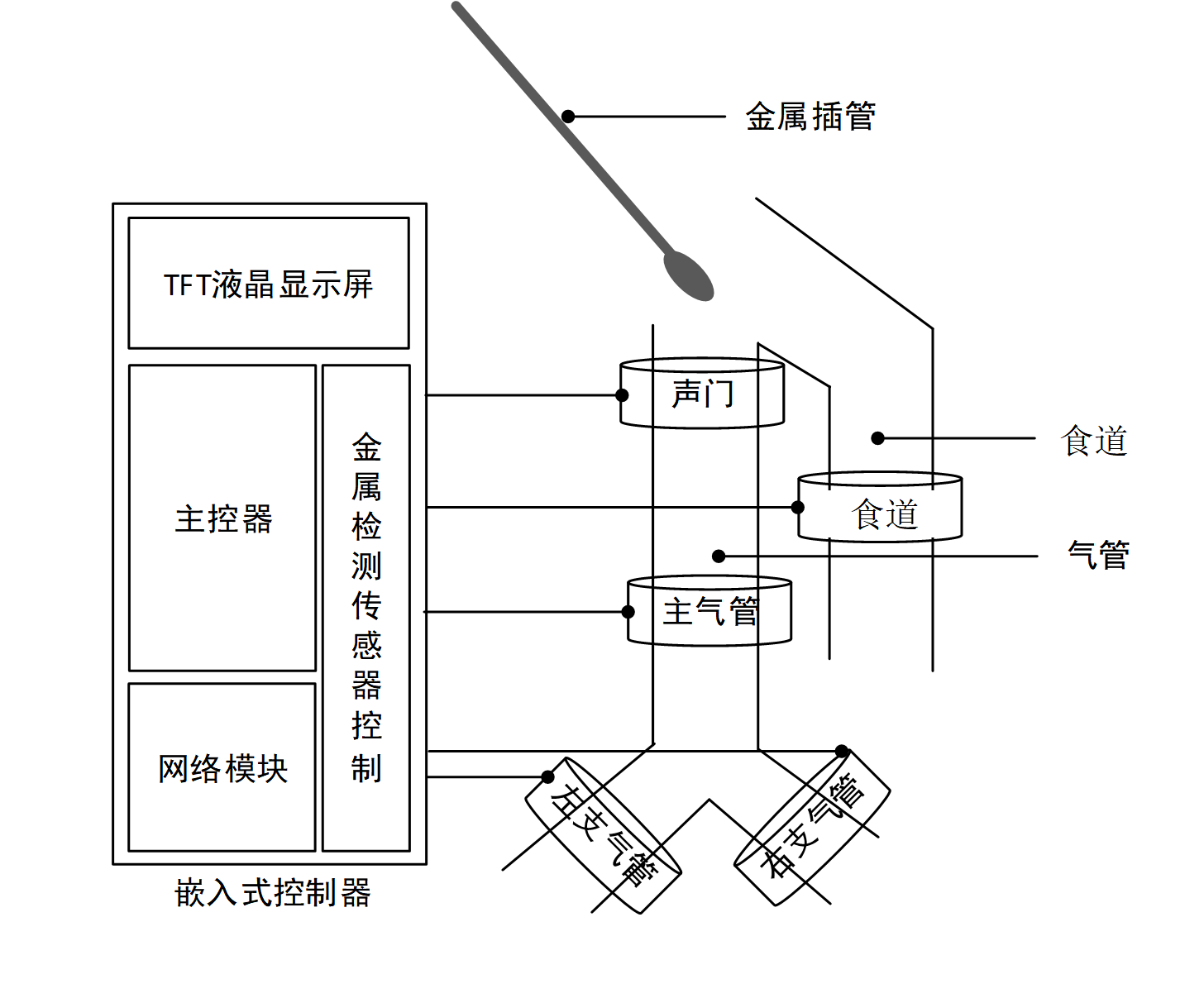


**Figure 1**

| Figure 1 translation from Chinese to English | |
| --- | --- |
| 金属插管 | Metal Intubation Tube |
| TFT液晶显示屏 | TFT LCD Display |
| 主控器 | Main Controller |
| 金属检测传感器 | Metal Detection Sensor |
| 网络模块 | Network Module |
| 嵌入式控制器 | Embedded Controller |
| 声门 | Glottis |
| 食道 | Esophagus |
| 气管 | Trachea |
| 主气管 | Main Trachea |

**Figure 2**

| Figure 2 translation from Chinese to English | |
| --- | --- |
| 启动摄像头，将摄像头角度调整到合适角度，能够完整拍摄到操作区域 | Start the camera and adjust its angle to properly capture the entire operating area |
| 登录训练平台，选择气管插管训练项目，查看摄像头拍摄是否正常 | Log in to the training platform, select the tracheal intubation training program, and check if the camera feed is normal |
| 在模拟人的声门、主气管、左支气管、右支气管和食管处安装好金属检测传感器，传感器连接到嵌入式控制器，打开嵌入式控制器的开关 | Install metal detection sensors at the glottis, main trachea, left bronchus, right bronchus, and esophagus of the simulator. Connect the sensors to the embedded controller and turn it on |
| 新网络环境 手动重置 | New network environment Manual reset |
| 重置嵌入式系统状态，使用手机连接嵌入式控制器的AP，设置要连接的WIFI账号和密码，设置成功后控制器将连接WIFI网络和服务器 | Reset the embedded system status. Use a mobile phone to connect to the embedded controller's AP, set up the WIFI account and password. After successful configuration, the controller will connect to the WIFI network and server |
| 读取上一次保存的WIFI账号和密码，连接WIFI网络和服务器 | Read the previously saved WIFI account and password to connect to the WIFI network and server |
| 否 | No |
| 连接服务成功 | Service connection successful |
| 是 | Yes |
| 线下模拟操作，金属传感器检测插管当前位置，嵌入式系统与服务端交互 | Offline simulation operation: metal sensors detect the current position of the tube, embedded system interacts with the server |
| 三维插管动画更新，数据统计更新 | 3D intubation animation updates, data statistics update |
| 操作结束，统计分析，得出成绩 | Operation completed, statistical analysis performed, results generated |

The invention discloses a training system and method for automatic evaluation of tracheal intubation operations based on the combination of embedded systems and three-dimensional visualization. The system includes a tracheal model and an esophageal model that simulate human anatomical structures. The tracheal model includes the glottis, main trachea, left bronchus, and right bronchus. Metal detection sensors are installed at the glottis, main trachea, left main bronchus, right main bronchus, and esophagus to detect whether metal passes through their respective channels. The sensors are connected to an embedded control system, which transmits the detected information to the embedded control system. The invention realizes the transmission of sensing data for key points such as position, force, depth, and speed during intubation, connects with the 3D simulation model for interactive visualization, and enables automatic evaluation, ultimately achieving quantifiable, standardized, and normalized operational assessments.

**Original version:**

**1.** 一种基于嵌入式和三维可视化结合的气管插管操作自动评估的训练系统，其特征在于，包括模拟人体结构的气管模型和食道模型，所述气管模型包括声门、主气管、左支气管和右支气管，在所述声门、主气管、左主支气管、右主支气管和食道位置安装有金属检测传感器，所述金属检测传感器用于检测是否有金属经过其所在部分的通道，并且金属检测传感器连接嵌入式控制系统，将检测到的信息发送至嵌入式控制系统。

**2.** 根据权利要求1所述的一种基于嵌入式和三维可视化结合的气管插管操作自动评估的训练系统，其特征在于，所述嵌入式控制系统包括主控制器、传感器控制模块、网络模块和显示模块。

**3.** 根据权利要求2所述的一种基于嵌入式和三维可视化结合的气管插管操作自动评估的训练系统，其特征在于，所述嵌入式控制器通过WIFI网络连接到后台服务器进行数据交互。

**4.** 根据权利要求1所述的一种基于嵌入式和三维可视化结合的气管插管操作自动评估的训练系统，其特征在于，在进行气管插管模拟操作时，通过金属检测传感器检测并对数据进行采集与记录，系统分析金属导管通过哪个部分的金属检测传感器，如果通过声门、气管则判定结果为正确，如果通过主支气管则判定结果未插入过深，如果通过食道则判定结果为错误。

**5.** 根据权利要求1所述的一种基于嵌入式和三维可视化结合的气管插管操作自动评估的训练系统，其特征在于，所述金属检测传感器通过检测电感变化来判断是否被触发，从而确定插管所在的位置；所述嵌入式控制系统通过引脚中断事件得到金属检测传感器被触发的消息，并将插管位置数据传输到服务器，所述服务器控制三维应用播放不同的动画，实现直观、及时的效果展示。

**6.** 根据权利要求1所述的一种基于嵌入式和三维可视化结合的气管插管操作自动评估的训练系统，其特征在于，使用塑料材质模拟声门、气管、主支气管以及食道，使用硅胶材质模拟皮肤肌肉组织，整体架构模拟人体基本结构。

**7.** 一种基于嵌入式和三维可视化结合的气管插管操作自动评估的训练方法，其特征在于，训练步骤为：

步骤1，启动摄像头，将摄像头角度调整到合适角度，能够完整拍摄到操作区域；

步骤2，打开web端程序，以账号和密码登录气管插管三维训练平台，登录后检查摄像头拍摄是否正常；

步骤3，打开气管插管模拟人装置上的嵌入式控制器；

步骤4，完成以上的操作后点击页面上的开始按钮然后进行训练，在操作时安装在模拟人声门、气管、左主支气管、右主支气管和食道的金属检测传感器会检测到金属插管的所在位置，之后嵌入式控制器通过网络将当前信息传输到后台服务器；

步骤5，每次训练结束后，进行数据的统计记录；自动保存训练的视频，用于后期回放。

**8.** 根据权利要求7一种基于嵌入式和三维可视化结合的气管插管操作自动评估的训练方法，其特征在于，通过区分模型材质和气管插管操作使用的导管材质，营造使用金属检测传感器检测出导管的条件。

**9.** 根据权利要求7一种基于嵌入式和三维可视化结合的气管插管操作自动评估的训练方法，其特征在于，通过使用金属检测传感器检测操作的导管从而分辨并判定该导管所在模型的具体位置。

**10.** 根据权利要求9一种基于嵌入式和三维可视化结合的气管插管操作自动评估的训练方法，其特征在于，根据操作的结果分以下判别标准：（1）声门触发：说明插管开始进入主气管，正常操作；（2）气管触发：插管位于主气管，正常操作；（3）左主支气管、右主支气管触发：插管通过了主气管，进入了支气管，是过度操作；（4）食道触发：插管没有进入主气管，而是进入了食道，是错误操作。

**一种基于嵌入式和三维可视化结合的气管插管操作自动评估的训练系统及方法**

**技术领域**

本发明涉及医学教育实训操作领域，具体涉及一种基于嵌入式和三维可视化结合的气管插管操作自动评估的训练系统及方法。

**背景技术**

传统的气管插管训练过程的判定是由专业教师现场评估。通过教师的经验判断学员插管的位置、力度、深度、速度是否正确，然而这样的训练方式存在下列5个问题：（1）对教师及教师的经验依赖程度高；（2）难以对实操训练效果标准量化；（3）训练枯燥乏味，训练的模型没有互动，学生的积极性不高；（4）只能课上训练，受场地、时间的限制，每个学生并不能都能得到教师的课上指导。（5）对于气管插管，由于有模型的遮盖，教师无法直接判断学员操作的正确性。

随着科技进步、三维仿真技术的成熟、嵌入式的模块化等多个学科融合得到关注，其在医学教育实训领域有极大的应用潜力。然而，目前缺乏一种能够通过三维仿真技术及嵌入想结合的训练系统。

**发明内容**

本发明的目的在于提供一种基于嵌入式和三维可视化结合的气管插管操作自动评估的训练系统及方法，提高训练效率和效果。

为实现上述目的，本发明采取的技术方案如下：

一方面，本发明提供一种基于嵌入式和三维可视化结合的气管插管操作自动评估的训练系统，其包括模拟人体结构的气管模型和食道模型，所述气管模型包括声门、主气管、左支气管和右支气管，在所述声门、主气管、左主支气管、右主支气管和食道位置安装有金属检测传感器，所述金属检测传感器用于检测是否有金属经过其所在部分的通道，并且金属检测传感器连接嵌入式控制系统，将检测到的信息发送至嵌入式控制系统。

进一步的，所述嵌入式控制系统包括主控制器、传感器控制模块、网络模块和显示模块。

更进一步的，所述嵌入式控制器通过WIFI网络连接到后台服务器进行数据交互。

进一步的，在进行气管插管模拟操作时，通过金属检测传感器检测并对数据进行采集与记录，系统分析金属导管通过哪个部分的金属检测传感器，如果通过声门、气管则判定结果为正确，如果通过主支气管则判定结果未插入过深，如果通过食道则判定结果为错误。

进一步的，所述金属检测传感器通过检测电感变化来判断是否被触发，从而确定插管所在的位置；所述嵌入式控制系统通过引脚中断事件得到金属检测传感器被触发的消息，并将插管位置数据传输到服务器，所述服务器控制三维应用播放不同的动画，实现直观、及时的效果展示。

进一步的，使用塑料材质模拟声门、气管、主支气管以及食道，使用硅胶材质模拟皮肤肌肉组织，整体架构模拟人体基本结构。

另一方面，本发明还提供一种基于嵌入式和三维可视化结合的气管插管操作自动评估的训练方法，训练步骤为：

步骤1，启动摄像头，将摄像头角度调整到合适角度，能够完整拍摄到操作区域；

步骤2，打开web端程序，以账号和密码登录气管插管三维训练平台，登录后检查摄像头拍摄是否正常；

步骤3，打开气管插管模拟人装置上的嵌入式控制器；

步骤4，完成以上的操作后点击页面上的开始按钮然后进行训练，在操作时安装在模拟人声门、气管、左主支气管、右主支气管和食道的金属检测传感器会检测到金属插管的所在位置，之后嵌入式控制器通过网络将当前信息传输到后台服务器；

步骤5，每次训练结束后，进行数据的统计记录；自动保存训练的视频，用于后期回放。

进一步的，通过区分模型材质和气管插管操作使用的导管材质，营造使用金属检测传感器检测出导管的条件。

进一步的，通过使用金属检测传感器检测操作的导管从而分辨并判定该导管所在模型的具体位置。

更进一步的，根据操作的结果分以下判别标准：（1）声门触发：说明插管开始进入主气管，正常操作；（2）气管触发：插管位于主气管，正常操作；（3）左主支气管、右主支气管触发：插管通过了主气管，进入了支气管，是过度操作；（4）食道触发：插管没有进入主气管，而是进入了食道，是错误操作。

本发明的有益效果是：本发明使得医护人员可以直观的判断自己的操作是否正确和标准，从而达到训练的目的。本系统实现了插管所在关键位置的检测，以及本系统可与三维可视化进行结合，实现直观、及时的效果展示。

**附图说明**

图1是本发明气管插管操作自动评估的训练系统原理图；

图2是本发明气管插管操作自动评估的训练方法流程图。

**具体实施方式**

下面结合附图和具体实施例对本发明进行详细说明。

实施例一

如图1，一种基于嵌入式和三维可视化结合的气管插管操作自动评估的训练系统，包括模拟人体结构的气管模型和食道模型，所述气管模型包括声门、主气管、左支气管和右支气管，其中使用塑料材质模拟声门、气管、主支气管以及食道，使用硅胶材质模拟皮肤肌肉组织，整体架构模拟人体基本结构；并且在所述声门、主气管、左主支气管、右主支气管和食道位置安装有金属检测传感器，所述金属检测传感器用于检测是否有金属经过其所在部分的通道，并且金属检测传感器连接嵌入式控制系统，将检测到的信息发送至嵌入式控制系统。

所述嵌入式控制系统包括主控制器、传感器控制模块、网络模块和显示模块。所述嵌入式控制器通过WIFI网络连接到后台服务器进行数据交互。在进行气管插管模拟操作时，通过金属检测传感器检测并对数据进行采集与记录，系统分析金属导管通过哪个部分的金属检测传感器，如果通过声门、气管则判定结果为正确，如果通过主支气管则判定结果未插入过深，如果通过食道则判定结果为错误。

所述金属检测传感器通过检测电感变化来判断是否被触发，从而确定插管所在的位置；所述嵌入式控制系统通过引脚中断事件得到金属检测传感器被触发的消息，并将插管位置数据传输到服务器，所述服务器控制三维应用播放不同的动画，实现直观、及时的效果展示。

实施例二

如图2，一种基于嵌入式与三维可视化结合的气管插管操作自动评估的训练方法，包括如下步骤：

步骤1，训练记录摄像头准备，启动摄像头，将摄像头角度调整到合适角度，能够完整拍摄到操作区域；

步骤2，打开web端程序，输入账号和密码登录到训练平台。进入三维气管插管训练平台后可以看到摄像头拍摄画面和气管插管初始的三维动画状态。

步骤3，打开气管插管模拟人装置上的嵌入式控制器，首次使用时需要长按上面的按钮进行重置设置，具体步骤为：

长按重置按钮五秒进行入重置程序；

打开手机上的WIFI，此时会搜索到一个叫ESP_ETT的热点网络，点击该网络进行连接操作；

连接成功后打开手机上的浏览器，输入http://esp_ett.local访问页面，此时会弹出网络配置页面，输入可用的WIFI名（不能有中文）和密码，以及部署的SOCKET服务地址，点击提交，此时气管插管的嵌入式系统就会主动连接WIFI和后台服务；

嵌入式控制器搭载的TFT液晶显示屏会显示系统的运行状态，当嵌入式系统无法与服务器建立正常的网络连接时需要检测当前网络环境或者进行重新设置操作，确保后续步骤能够正常进行。

步骤4，完成以上的操作后点击页面上的开始按钮就可以进行训练了，在操作时会根据插入的气管位置进行传感器识别并且会在网页上播放动画，同时显示插入到的部位。

在操作中，通过在塑料、硅胶模型仿真声门、气管、左右主支气管、食道内壁嵌入式的感应模块和气管插管金属材质内芯两部分，金属检测传感器在感应金属经过该部分通道时可进行提示，将该传感器连接到嵌入式控制系统，嵌入式控制器通过WIFI网络连接到后台服务器进行数据交互。在进行气管插管模拟操作时，通过金属检测传感器检测提示数据采集与记录，分析金属导管通过哪些金属检测传感器。金属检测传感器根据检测到电感的变化判断是否被触发；嵌入式系统利用引脚的中断特性在金属检测传感器输出电平发生变化时更新检测状态，并将当前变化数据通过之前建立的网络传输给后台服务器；三维仿真程序事先制作了声门、气管、左主支气管、右主支气管和食道不同部位被触发对应的动画，当嵌入式系统通知状态更新时三维仿真程序就播放对应的动画，实现直观、及时的训练效果展示。

根据操作的结果分以下判别标准：（1）声门触发：说明插管开始进入主气管，正常操作；（2）气管触发：插管位于主气管，正常操作；（3）左主支气管、右主支气管触发：插管通过了主气管，进入了支气管，是过度操作；（4）食道触发：插管没有进入主气管，而是进入了食道，是错误操作。

步骤5，每次训练结束后，进行数据的记录保存；训练的视频会自动保存，可以进行回放。

上述流程中，实现了插管所在关键位置的检测：步骤4使用金属插管和金属检测传感器配合，当金属插管穿过安装在声门、气管、左主支气管、右主支气管和食道的金属检测传感器时会引起检测传感器的电感变化，利用嵌入式控制器的引脚中断捕获到传感器的触发状态，从而确定插管所在位置。

上述流程中，实现了嵌入式系统与三维可视化的结合：步骤4使用金属检测传感器和嵌入式控制器获得气管插管的操作状态，通过网络传输方式和三维应用进行交互，三维应用播放不同的操作动画。

综上，本发明实现基于插管过程中的位置、力度、深度、速度等由人眼不能直接观察到的关键点的感应数据传输，并和三维仿真模型连通互动，将操作过程以可视化的方式展示，并可根据位置、力度、深度、速度等考核评估点实现自动评估，以最终实现操作考核的可量化、标准化和规范化。

以上显示和描述了本发明的基本原理、主要特征和优点。本领域的普通技术人员应该了解，上述实施例不以任何形式限制本发明的保护范围，凡采用等同替换等方式所获得的技术方案，均落于本发明的保护范围内。

本发明未涉及部分均与现有技术相同或可采用现有技术加以实现。


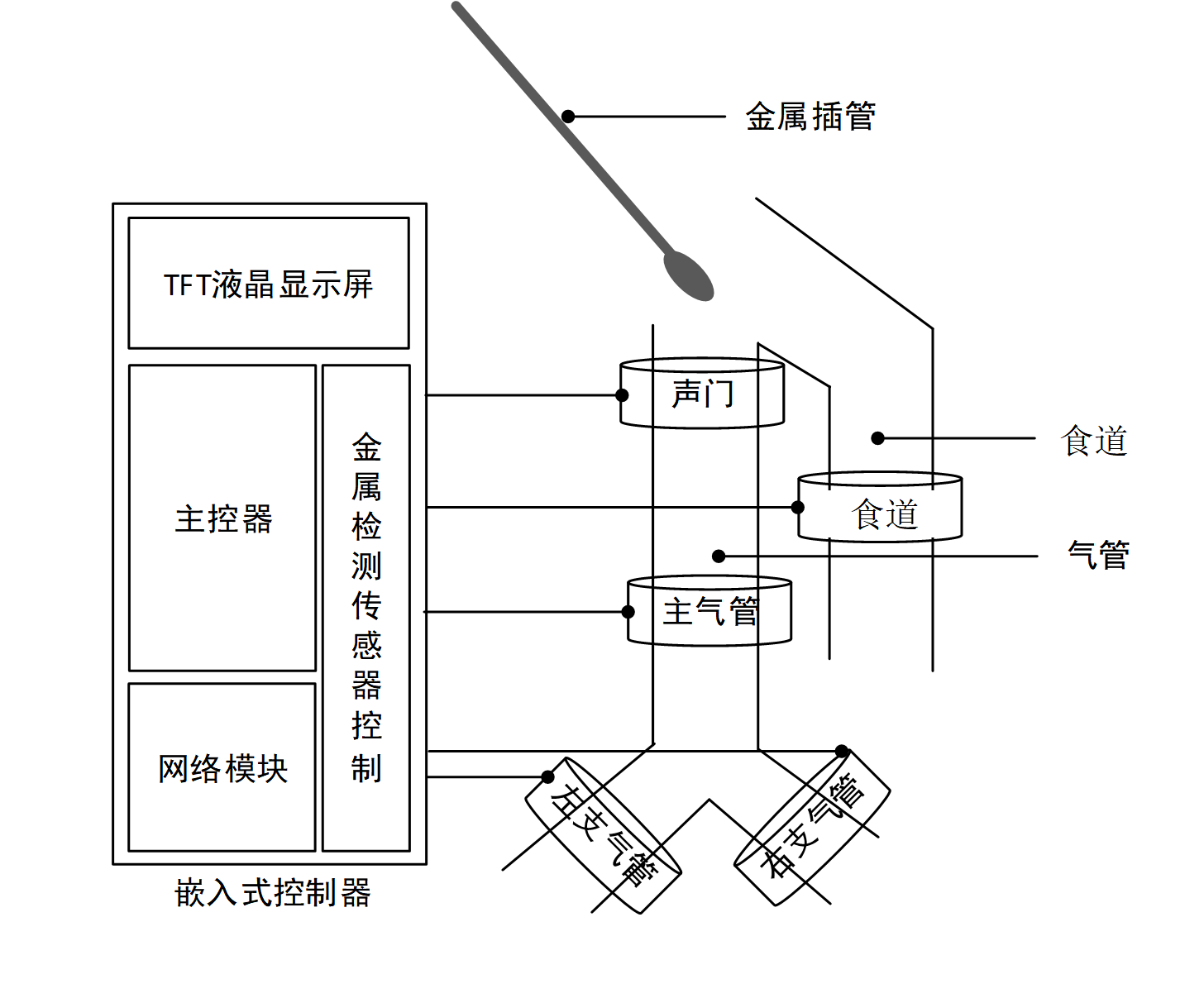


图1

图2

本发明公开了一种基于嵌入式和三维可视化结合的气管插管操作自动评估的训练系统及方法，所述系统包括模拟人体结构的气管模型和食道模型，所述气管模型包括声门、主气管、左支气管和右支气管，在所述声门、主气管、左主支气管、右主支气管和食道位置安装有金属检测传感器，所述金属检测传感器用于检测是否有金属经过其所在部分的通道，并且金属检测传感器连接嵌入式控制系统，将检测到的信息发送至嵌入式控制系统。本发明实现基于插管过程中的位置、力度、深度、速度等由人眼不能直接观察到的关键点的感应数据传输，并和三维仿真模型连通互动，将操作过程以可视化的方式展示，并可实现自动评估，以最终实现操作考核的可量化、标准化和规范化。
