# Supplementary material for: Can artificial intelligence outperform experts in assessing clinical skills? Evidence from a comparative experiment
Source: Front Med (Lausanne). 2026 Jun 8;13:1847867. doi: 10.3389/fmed.2026.1847867 (PMC13283888; doi:10.3389/fmed.2026.1847867)
Supplement: Supplementary file 1 [file Data_Sheet_1.zip › supplement/1. AIA systems introduction/history-taking AI assessment system introduction.docx]

Translation version：

Technical Pathway of Virtual Standardized Patient (VSP) Diagnosis and Treatment Training System

The VSP training system primarily revolves around core technologies including multimodal interaction, artificial intelligence, virtual reality, and medical knowledge modeling. It aims to digitally simulate real patients' symptoms, signs, and interactive behaviors, providing medical students or physicians with an immersive and repeatable clinical training environment. Below is a concise overview of its key technical pathways:

1. System Architecture Design

Modular Layered Architecture:

Includes user interaction layer (UI/VR/AR interfaces), logic processing layer (AI engine), medical knowledge base layer (symptom-disease association database), and evaluation feedback layer (automated scoring system).

Cloud Platform or Local Deployment:

Supports multi-terminal access (PC/VR/mobile devices) and ensures low-latency, high-concurrency training.

2. Core Technical Modules

(1) Virtual Patient Modeling

Physiological & Pathological Models:

Dynamic mathematical models (e.g., finite-state machines or agent-based models) built using physiological parameters (e.g., heart rate, blood pressure) and disease-specific features (e.g., cough frequency in pneumonia).

Appearance & Behavior Simulation:

High-fidelity virtual humans created with 3D modeling tools (Maya), combined with motion capture to simulate signs like pain or dyspnea.

(Maya is used for scenarios such as 3D modeling, animation rendering, and visual creation in film, television, and games. Address: https://www.autodesk.com/products/maya/free-trial)

(2) Natural Language Interaction (NLP)

Speech Recognition & Synthesis:

ASR (e.g., Whisper) converts user speech to text; TTS (e.g., Microsoft) generates virtual patient responses.

(Whisper is an open-source speech recognition model developed by OpenAI, supporting multilingual speech-to-text conversion, meeting transcription, subtitle generation, and other applications. Address: <https://whisperai.com/)>

(We are using Microsoft's commercial text-to-speech service. Address: https://learn.microsoft.com/zh-cn/azure/ai-services/speech-service/)

Intent Understanding & Dialogue Management:

Dialogue trees based on medical knowledge graphs or dynamic responses generated via LLMs (e.g., doubao-seed), supporting open-ended consultations.

(3) Multimodal Sign Simulation

Haptic Feedback:

Force feedback devices simulate abdominal palpation or pulse detection.

Visual/Auditory Feedback:

Rendered signs like jaundice or auscultation sounds (e.g., heart sound synthesis algorithms), enhanced by VR headsets or mixed reality (MR) devices.

(4) Medical Knowledge Base & Reasoning

Knowledge Graph Construction:

Integrates clinical guidelines (e.g., UpToDate) and real case data to establish symptom-disease-examination-treatment networks.

Diagnostic Reasoning Engine:

Rule-based systems (e.g., Drools) or machine learning (e.g., Bayesian networks) simulate clinical decision-making, with dynamic condition adjustments (e.g., complication triggers).

3. Training & Evaluation Mechanisms

Automated Scoring System:

Analyzes user actions (e.g., consultation sequence, test appropriateness) via logs and generates evaluations against standard pathways (e.g., SOAP workflow).

Real-Time Feedback & Guidance:

Provides error correction (e.g., missed key questions) or adaptive difficulty (e.g., increased case complexity).

4. Technical Challenges & Solutions

Authenticity Limitations:

High-precision AI models trained on medical data (e.g., EMR-based symptom generation), refined via expert review.

Computational Demands:

Edge computing reduces rendering latency; lightweight models enable mobile deployment.

Privacy & Compliance:

Anonymized training data adhering to HIPAA/GDPR regulations.

5. Application Expansion

Cross-Disciplinary Integration:

Supports nursing, emergency care (e.g., virtual ICU), and other scenarios.

Remote Collaboration:

Enables multi-user consultations (e.g., WebRTC-based virtual medical teams).

Conclusion

The VSP system’s technical pathway centers on an "interaction-simulation-reasoning-evaluation" closed loop, relying on deep integration of AI, VR, and medical knowledge. Future advancements may leverage generative AI (e.g., Diffusion models for synthetic cases) to further enhance personalization and dynamism.

Technical Solution for Medical History Collection Scoring Mechanism

**1.Scoring Dimension Design (Multi-dimensional Quantitative Scoring, Total 100 Points)**

| **Scoring Dimension** | **Weight** | **Description** |
| --- | --- | --- |
| History Taking Assessment | 70% | Based on standardized checklist |
| History Taking Skills | 10% | Logical organization, prioritization, sequence of inquiry, communication skills, and use of complex terminology |
| Humanistic Care | 10% | Assessment of empathy and patient-centered communication |
| Medical Record Writing | 10% | Evaluation of documentation quality |

**2. History Taking Checklist**

**2.1 Example of mandatory items** (using a chest pain case):

General Information: Patient's basic information (5 points); Present Illness: Pain location (2 points); Present Illness: Pain characteristics (2 points); Present Illness: Pain duration (2 points); Present Illness: Precipitating/relieving factors (1 point); Past Medical History: Cardiovascular disease history (5 points); Past Medical History: Allergy history (5 points); Personal History: Smoking history (5 points); Family History: Premature coronary artery disease history (5 points).

**2.2 Scoring Logic:**

Student asks and obtains the corresponding information → Full points awarded

Key mandatory item omitted → No points awarded

Item asked but information obtained is inaccurate → Partial points awarded

**3. Dual-Layer Mechanism: "Rule Engine + LLM-Assisted Verification"**

**3.1 First Layer: Rule Engine (Objective Scoring, 70% weight)**

Semantic understanding: Extract key medical entities from the student's history-taking text

Slot filling: Verify whether mandatory information slots are complete

Example: Determine whether the key feature "pain radiating to the left shoulder" has been elicited

**3.2 Second Layer: LLM Semantic Evaluation (Subjective Scoring, 30% weight)**

Using the **DOUBAO-SEED** **BGE-M3 Embedding model** to evaluate:

Semantic similarity between the history-taking text and the standard medical history (threshold ≥ 0.75 considered passing)

Using the **DOUBAO-SEED large language model** to evaluate:

Information coverage completeness score

Logical coherence of the history-taking process

Note: This system does not employ a self-developed foundational large language model. Instead, it utilizes ByteDance's "Doubao" large language model (Doubao-SEED) as its natural language understanding engine. Literature indicates that Doubao-SEED has passed the Chinese National Medical Licensing Examination.（https://doi.org/10.2196/77978）

(Doubao-SEED Address: https://seed.bytedance.com/en/)

**4. Scoring Process Architecture**

Student voice or text input → ASR (Automatic Speech Recognition) → Text

[Rule Engine] Keyword extraction + Slot filling → Objective score

[LLM Evaluation] Semantic similarity + Completeness assessment → Subjective score

Comprehensive score (0–100 points) + Feedback on missing items

Generation of detailed scoring report (brief explanation of scoring rationale)

**5.Threshold Settings**

| **Indicator** | **Threshold** | **Description** |
| --- | --- | --- |
| Semantic Similarity | ≥ 0.75 | Calculated based on the BGE-M3 Embedding model |
| Mandatory Item Coverage | ≥ 80% | Minimum passing threshold |
| Information Completeness | ≥ 85% | Excellence threshold |

Original version:

虚拟标准化病人（VSP）诊疗训练系统的技术路径主要围绕多模态交互、人工智能、虚拟现实和医学知识建模等核心技术展开，旨在通过数字化手段模拟真实患者的症状、体征和交互行为，为医学生或医生提供沉浸式、可重复的临床训练环境。以下是其关键技术路径的简要概述：

1. 系统架构设计

模块化分层架构：

包括用户交互层（UIVRAR界面）、逻辑处理层（AI引擎）、医学知识库层（症状-疾病关联数据库）、评估反馈层（自动化评分系统）等。

云平台或本地部署：支持多终端（PCVR移动设备）访问，确保低延迟和高并发训练。

2. 核心技术模块

（1）虚拟病人建模

生理与病理模型：

基于生理学参数（如心率、血压）和疾病特征（如肺炎的咳嗽频率），构建动态数学模型（如有限状态机或基于代理的模型）。

外观与行为仿真：

使用3D建模工具（Maya）生成高保真虚拟人，结合动作捕捉（Motion Capture）模拟疼痛、呼吸困难等体征。

（2）自然语言交互（NLP）

语音识别与合成：

通过ASR（如Whisper）转换用户语音为文本，TTS（如Microsoft）生成虚拟病人的语音回应。

意图理解与对话管理：

基于医学知识图谱构建对话树，或采用大语言模型（如doubao-seed）生成动态响应，支持开放式问诊。

（3）多模态体征模拟

触觉反馈：

通过力反馈设备模拟腹部触诊、脉搏触感等。

视觉听觉反馈：

渲染皮肤黄疸、听诊音（如心音合成算法）等，结合VR头显或混合现实（MR）设备增强沉浸感。

（4）医学知识库与推理

知识图谱构建：

整合临床指南（如UpToDate）、真实病例数据，建立症状-疾病-检查-治疗的关联网络。

诊断推理引擎：

基于规则系统（Drools）或机器学习（如贝叶斯网络）模拟临床思维，动态调整病情（如并发症触发）。

3. 训练与评估机制

自动化评分系统：

通过日志分析用户操作（如问诊顺序、检查合理性），结合标准诊疗路径（如SOAP流程）生成评估报告。

实时反馈与引导：

提供错误纠正提示（如遗漏关键问诊项）或自适应难度调整（如病情复杂化）。

4. 技术挑战与解决方案

真实性瓶颈：

通过高精度医学数据训练AI模型（如基于EMR数据的症状生成），结合专家审核迭代优化。

算力需求：

采用边缘计算降低渲染延迟，或使用轻量化模型部署在移动端。

隐私与合规：

匿名化训练数据，符合HIPAAGDPR等医疗数据规范。

5. 应用扩展

跨学科整合：

支持护理、急救等多场景训练（如虚拟ICU）。

远程协作：

支持多用户协同会诊（如基于WebRTC的虚拟医疗团队）。

总结

虚拟标准化病人系统的技术路径以“交互-仿真-推理-评估”为核心闭环，依赖AI、VR与医学知识的深度耦合，未来可能通过生成式AI（如Diffusion模型合成病例）进一步突破个性化与动态化限制。
